# Biallelic mutations in neurofascin cause neurodevelopmental impairment and peripheral demyelination

**DOI:** 10.1093/brain/awz248

**Published:** 2019-09-09

**Authors:** Stephanie Efthymiou, Vincenzo Salpietro, Nancy Malintan, Mallory Poncelet, Yamna Kriouile, Sara Fortuna, Rita De Zorzi, Katelyn Payne, Lindsay B Henderson, Andrea Cortese, Sateesh Maddirevula, Nadia Alhashmi, Sarah Wiethoff, Mina Ryten, Juan A Botia, Vincenzo Provitera, Markus Schuelke, Jana Vandrovcova, SYNAPS Study Group, Stanislav Groppa, Blagovesta Marinova Karashova, Wolfgang Nachbauer, Sylvia Boesch, Larissa Arning, Dagmar Timmann, Bru Cormand, Belen Pérez-Dueñas, Jatinder S Goraya, Tipu Sultan, Jun Mine, Daniela Avdjieva, Hadil Kathom, Radka Tincheva, Selina Banu, Mercedes Pineda-Marfa, Pierangelo Veggiotti, Michel D Ferrari, Arn M J M van den Maagdenberg, Alberto Verrotti, Giangluigi Marseglia, Salvatore Savasta, Mayte García-Silva, Alfons Macaya Ruiz, Barbara Garavaglia, Eugenia Borgione, Simona Portaro, Benigno Monteagudo Sanchez, Richard Boles, Savvas Papacostas, Michail Vikelis, James Rothman, Dimitri Kullmann, Eleni Zamba Papanicolaou, Efthymios Dardiotis, Shazia Maqbool, Shahnaz Ibrahim, Salman Kirmani, Nuzhat Noureen Rana, Osama Atawneh, Shen-Yang Lim, Farooq Shaikh, George Koutsis, Marianthi Breza, Salvatore Mangano, Carmela Scuderi, Eugenia Borgione, Giovanna Morello, Tanya Stojkovic, Massimo Zollo, Gali Heimer, Yves A Dauvilliers, Carlo Minetti, Issam Al-Khawaja, Fuad Al-Mutairi, Sherifa Hamed, Menelaos Pipis, Conceicao Bettencourt, Simon Rinaldi, Laurence Walsh, Erin Torti, Valeria Iodice, Maryam Najafi, Ehsan Ghayoor Karimiani, Reza Maroofian, Karine Siquier-Pernet, Nathalie Boddaert, Pascale De Lonlay, Vincent Cantagrel, Mhammed Aguennouz, Mohamed El Khorassani, Miriam Schmidts, Fowzan S Alkuraya, Simon Edvardson, Maria Nolano, Jérôme Devaux, Henry Houlden

**Affiliations:** 1 Department of Neuromuscular Disorders, UCL Institute of Neurology, Queen Square, London, UK; 2 Department of Clinical and Experimental Epilepsy, UCL Institute of Neurology, Queen Square, London, UK; 3 INSERM U1051, Institut de Neurosciences de Montpellier (INM), Université de Montpellier, Montpellier, France; 4 Unit of Neuropediatrics and Neurometabolism, Pediatric Department 2, Rabat Children’s Hospital, and Faculty of Medicine and Pharmacy of Rabat, University Mohammed V of Rabat, Morocco; 5 Department of Chemical and Pharmaceutical Sciences, University of Trieste, Trieste, Italy; 6 Riley Hospital for Children, Indianapolis, Indiana, IN, USA; 7 GeneDx, Gaithersburg, MD, USA; 8 Department of Genetics, King Faisal Specialist Hospital and Research Centre, Riyadh, Saudi Arabia; 9 Department of Genetics, College of Medicine, Sultan Qaboos University, Sultanate of Oman; 10 Center for Neurology and Hertie Institute for Clinical Brain Research, Eberhard Karls-University, Tübingen, Germany; 11 Department of Neurodegenerative Diseases, UCL Institute of Neurology, Queen Square, London, UK; 12 Departamento de Ingeniería de la Información y las Comunicaciones, Universidad de Murcia, Murcia, E, Spain; 13 Department of Neurology, Istituti Clinici Scientifici Maugeri IRCCS, Italy; 14 Department of Neuropediatrics, Charité Universitätsmedizin Berlin, Germany; 15 Department of Brain Repair and Rehabilitation, Institute of Neurology, University College London, UK; 16 Autonomic Unit, National Hospital Neurology and Neurosurgery, UCL NHS Trust, London, UK; 17 Genome Research Division, Human Genetics Department, Radboud University Medical Center, Geert Grooteplein Zuid 10, Nijmegen, The Netherlands; 18 Genetics Research Centre, Molecular and Clinical Sciences Institute, St George’s, University of London, Cranmer Terrace, London, UK; 19 Paris Descartes - Sorbonne Paris Cité University, Imagine Institute, Paris, France; 20 Developmental Brain Disorders Laboratory, Imagine Institute, INSERM UMR 1163, Paris, France; 21 Department of Pediatric Radiology, Necker Enfants Malades University Hospital, APHP, Paris, France; 22 Inserm, U1151, Institut Necker-Enfants Malades, Paris, France; 23 Department of Clinical and Experimental Medicine, University of Messina, Sicily; 24 Center for Pediatrics and Adolescent Medicine, University Hospital Freiburg, Freiburg University, Faculty of Medicine, Mathildenstrasse 1, Freiburg, Germany; 25 Department of Anatomy and Cell Biology, College of Medicine, Alfaisal University, Riyadh, Saudi Arabia; 26 Paediatric Neurology Unit, Hadassah Medical Center, Jerusalem, Israel; 27 Department of Neurosciences, Reproductive and Odontostomatological Sciences, University “Federico II” of Naples, Italy

**Keywords:** neurofascin, neurodevelopment, peripheral demyelination

## Abstract

Axon pathfinding and synapse formation are essential processes for nervous system development and function. The assembly of myelinated fibres and nodes of Ranvier is mediated by a number of cell adhesion molecules of the immunoglobulin superfamily including neurofascin, encoded by the *NFASC* gene, and its alternative isoforms Nfasc186 and Nfasc140 (located in the axonal membrane at the node of Ranvier) and Nfasc155 (a glial component of the paranodal axoglial junction). We identified 10 individuals from six unrelated families, exhibiting a neurodevelopmental disorder characterized with a spectrum of central (intellectual disability, developmental delay, motor impairment, speech difficulties) and peripheral (early onset demyelinating neuropathy) neurological involvement, who were found by exome or genome sequencing to carry one frameshift and four different homozygous non-synonymous variants in *NFASC.* Expression studies using immunostaining-based techniques identified absent expression of the Nfasc155 isoform as a consequence of the frameshift variant and a significant reduction of expression was also observed in association with two non-synonymous variants affecting the fibronectin type III domain. Cell aggregation studies revealed a severely impaired Nfasc155-*CNTN1/CASPR1* complex interaction as a result of the identified variants. Immunofluorescence staining of myelinated fibres from two affected individuals showed a severe loss of myelinated fibres and abnormalities in the paranodal junction morphology. Our results establish that recessive variants affecting the Nfasc155 isoform can affect the formation of paranodal axoglial junctions at the nodes of Ranvier. The genetic disease caused by biallelic *NFASC* variants includes neurodevelopmental impairment and a spectrum of central and peripheral demyelination as part of its core clinical phenotype. Our findings support possible overlapping molecular mechanisms of paranodal damage at peripheral nerves in both the immune-mediated and the genetic disease, but the observation of prominent central neurological involvement in *NFASC* biallelic variant carriers highlights the importance of this gene in human brain development and function.

## Teaser

Neurofascin (*NFASC*) isoforms are immunoglobulin cell adhesion molecules involved in node of Ranvier assembly. Efthymiou *et al.* identify biallelic *NFASC* variants in ten unrelated patients with a neurodevelopmental disorder characterized by variable degrees of central and peripheral involvement. Abnormal expression of Nfasc155 is accompanied by severe loss of myelinated fibres.

See Karakaya and Wirth (doi:10.1093/brain/awz273) for a scientific commentary on this article.

## Introduction

**Figure 1:**
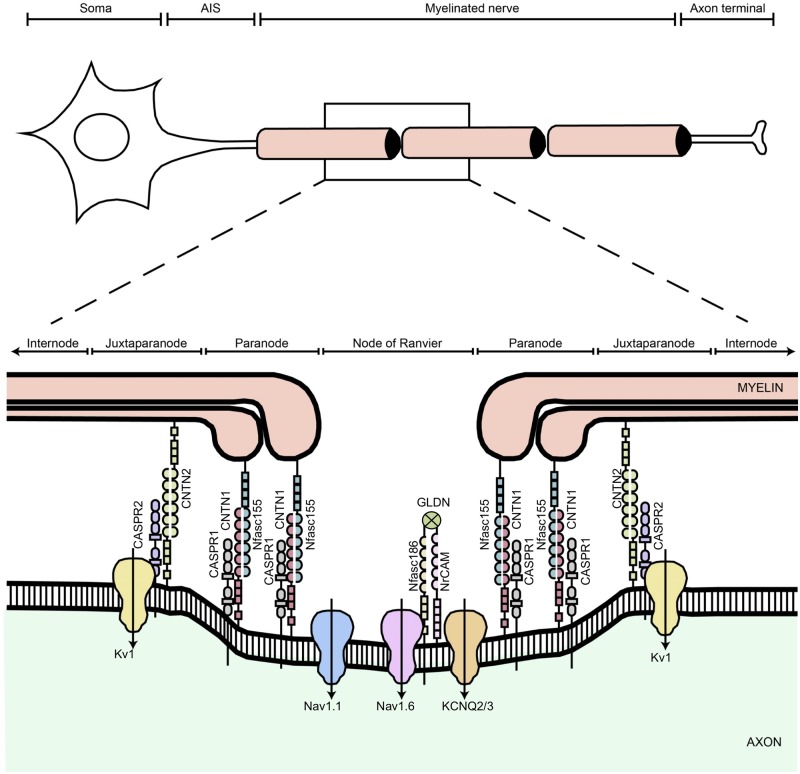
**Schematic diagram showing the different domains of a myelinated axon.** The axonal region around the node of Ranvier is expanded to show the different axonal domains: the node of Ranvier where voltage-gated Na^+^ channels (Na_v_1.6 and Na_v_1.1) are expressed, the paranode where the myelin is attached to the axon, and the juxtaparanode where most voltage-gated K^+^ channels (KCNQ2/3 and K_v_1) are located. Each of these domains is characterized by the expression of specific cell adhesion molecules; at the nodes Nfasc186 binds gliomedin (GLDN) and NrCAM, which are secreted by Schwann cells in the nodal gap lumen, at the paranode Nfasc155 forms a complex with CNTN1/CASPR1 to form the septate-like junctions, and at the juxtaparanode the CNTN2/CASPR2 complex enables the sequestration of K_v_1 channels. Adapted and modified from Arancibia-Carcamo and Attwell (2014).

In the vertebrate nervous system, myelinated fibres allow rapid nerve impulse transmission because of the ensheathment of their axons by specialized glial cells to form the multilamellar myelin sheath. Also, voltage-gated sodium channels (Na_v_) are clustered at the nodes of Ranvier further facilitating the rapid conduction of nerve impulses in the vertebrate brain and peripheral nerves (Ghosh *et al.*, 2018). Neurofascin protein isoforms Nfasc186 and Nfasc155 are splice variants encoded by the *NFASC *gene (MIM: 609145) and belong to the L1 family of immunoglobulin cell adhesion molecules, which play a critical role in the assembly of the node of Ranvier (Ghosh *et al.*, 2018). Nfasc155 is a glial isoform abundantly expressed in myelinating Schwann cells and predominantly enriched at the paranodal sites, acting as a ligand for the axonal contactin-1 (CNTN1)/contactin-associated protein-1 (CASPR1) complex. Nfasc186 represents the axonal isoform expressed at the node of Ranvier membranes where it is involved in the long-term maintenance and stability of Na_v_ channels (Fig. 1) (Sherman *et al.*, 2005; Taylor *et al.*, 2018). Autoantibodies against Nfasc186 and Nfasc155 are associated with severe forms of chronic inflammatory demyelinating polyneuropathy (CIDP) and cause an immune-mediated nodo-paranodopathy (Querol *et al.*, 2017; Vallat *et al.*, 2018) further demonstrating that these proteins play a crucial role in conduction. Biallelic variants in gliomedin (*GLDN*) and *CNTNAP1* encoding essential components of the nodes of Ranvier and paranodes, respectively, lead to inherited nodo-paranodopathies, a distinct disease entity among peripheral neuropathies (Maluenda *et al.*, 2016). Smigiel *et al.* (2018) report the identification of a homozygous truncating *NFASC* mutation affecting the fibronectin type III domain, specific to the Nfasc155 isoform in a child presenting with a very severe neurodevelopmental disorder resembling spinal muscular atrophy, while Monfrini *et al.* (2019) report a homozygous missense mutation in a case with autosomal recessive ataxia and a demyelinating neuropathy. Importantly, a human Mendelian disease caused by *NFASC* variants has been reported only in these two single individuals to date.

Here, we describe 10 individuals from six families carrying homozygous non-synonymous or frameshift variants in *NFASC.* Phenotypic analysis of affected individuals revealed, in all cases, global developmental delay and weakness with variable features of chronic demyelinating neuropathy in some cases. For some of the identified variants, we compared surface expression of the disease variant and wild-type Nfasc155 isoforms. We also tested the capability of variant proteins to interact with other partner proteins at paranodal sites, including gliomedin and the CNTN1/CASPR1 complex. Abnormalities of paranodal junction were identified by immunofluorescence analyses of myelinated fibres from skin samples of two affected individuals carrying homozygous variants in *NFASC.* Our results link biallelic variants in *NFASC* isoforms at glial cells to defects in the paranodal axoglial junctions and phenotypes that range from variable neurodevelopmental impairment to weakness, central hypomyelination, and peripheral chronic demyelinating neuropathy.

## Materials and methods

This study was approved by local institutional IRB/ethical review boards of all participating centres, and written informed consent was obtained prior to genetic testing from all the families involved. Clinical details were obtained through medical file review and clinical examination. Genomic DNA was extracted from peripheral blood samples according to standard procedures. Whole exome sequencing (WES) was performed as described elsewhere (Mencacci *et al.*, 2016), and our bioinformatics filtering strategy included screening for only exonic and donor/acceptor splicing variants. In accordance with the pedigree and phenotype, priority was given to rare variants [<0.01% in public databases, including 1000 Genomes project, NHLBI Exome Variant Server, Complete Genomics 69, and Exome Aggregation Consortium (ExAC v0.2)] that were fitting a recessive (homozygous or compound heterozygous) or a *de novo* model and/or variants in genes previously linked to neuropathy, intellectual disability and other neurological disorders. Other families were recruited through GeneMatcher (https://genematcher.org/).

### Molecular biology

NFASC missense constructs were cDNA synthesized by GenScript into pCMV-3Tag-4A by replacing the coding sequence for these inserts via BamHI and XhoI (NEB), giving rise to pCMV_Nfasc186[WT], pCMV_Nfasc186[N130D], pCMV_Nfasc186[R359P], pCMV_Nfasc186[P694T], pCMV_Nfasc186[S820P], pCMV_Nfasc155[WT], pCMV_Nfasc155[N124D], pCMV_Nfasc155[R370P], pCMV_Nfasc155[P705T], pCMV_Nfasc155[S831P] and pCMV_Nfasc155[P939Ter].

To generate pCMV_Nfasc155[P939Ter], the respective variant was introduced with the QuickChange Site-Directed Mutagenesis Kit (Agilent Technologies). A Myc epitope was then inserted before the stop codon into pCMV_Nfasc155[P939Ter] using site-directed mutagenesis kit with primers: 5′-GAACAAAAACT CATCTCAGAAGAGGATCTGtgattggatacactctcaaat-′3 and 5′-CAGATCCTCTTCTGAGATGAGTTTTTGTTCTGATCCCATTTGGATGCTCAG-3′.

### Cell assays

Human embryonic kidney (HEK) cells were transfected with Nfasc155 or Nfasc186 variants using JetPEI (Polyplus-transfection). After 48 h, cells were incubated for 20 min at 37°C with rabbit antibodies against Nfasc186 (D6G60; Cell Signaling Technology) or Nfasc155 (D7B6O; Cell Signaling Technology) diluted 1/50 in Opti-MEM™ or with 1 µg of gliomedin-Fc (Devaux, 2012) diluted in Opti-MEM™ (ThermoFisher Scientific). Cells were then washed in phosphate-buffered saline (PBS), fixed with 2% paraformaldehyde in PBS and blocked with blocking solution (5% fish skin gelatin containing, 0.1% Triton™ X-100 in PBS). HEK cells were incubated with a mouse anti-Myc antibody (Roche; 1/200) for 1 h, then incubated with Alexa conjugated secondary antibodies (1/500; Jackson ImmunoResearch) for 30 min. Coverslips were washed three times in PBS, stained with DAPI, and mounted with Mowiol® plus 2% DABCO. In some experiments, HEK cells were co-transfected with GFP and Nfasc155 variants. In those experiments, cells were fixed and revealed with a mouse anti-Myc antibody. Immunostainings were examined using an ApoTome fluorescence microscope (ApoTome, AxioObserver and AxioCam MRm, Carl Zeiss MicroImaging GmbH). Digital images were manipulated into figures with CorelDRAW and Corel Photo-Paint.

### Cell aggregation assay

N2A cells were plated in 6-well plates at a density of 500 000 cells/well and were transiently transfected using JetPEI (Polyplus-transfection) with Nfasc155 and ptdTomato-N1 (Clontech), CNTN1/CASPR1 and peGFP-N1 (Clontech), or peGFP-N1 alone. The day after, cells were trypsinized using 0.05% trypsin in PBS and suspended in 1 ml serum free Opti-MEM™ medium (ThermoFisher Scientific). Cells were mixed together in a 1:1 ratio (400 000 cells/ml), and then agitated at 100 rpm for 2 h at 37°C. Cell suspension (100 µl) was then mounted between slides and coverslip, and was immediately observed using a fluorescence microscope with 5× objective. The number of cells in mixed aggregates was evaluated in 10 fields. Aggregates were defined as clusters of at least four cells including red and green cells. The four experiments were performed for each condition.

### Immunoblots

HEK cells were transfected with Nfasc155 constructs for 24 h, then the cells were washed in PBS and solubilized on ice for 15 min in 1% Triton™ X-100, 140 mM NaCl, 20 mM Tris-HCl, pH 7.4 containing protease inhibitors. Proteins (50 µg) were loaded on 7.5% SDS-PAGE gels, transferred, and immunoblotted with a mouse antibody against Myc (1:2000) or a mouse antibody against α-tubulin (1:2000; MABT205; Merck). Immunoreactivity was revealed using peroxidase-coupled secondary antibodies (1:5000; Jackson ImmunoResearch) and BM chemiluminescence kit (Roche). The integrated densities of each protein band were measured with ImageLab software (Bio-Rad).

### Molecular modelling

Wild-type and mutated *NFASC* isoforms were built from structure PDB ID 3P3Y (Supplementary material). Each protein was placed in a cubic box and minimized. A water layer of 0.8 nm and Na^+^ ions to neutralize the system were added, and a second minimization was performed. In all cases we used AMBER99SB-ILDN (Lindorff-Larsen *et al.*, 2010) force field and simple point charge water. In all systems we performed NVT [constant number of atoms, volume and temperature (canonical ensemble)] and NPT [constant number of atoms, pressure and temperature (isothermal-isobaric ensemble)] equilibrations for 100 ps, followed by 360 ns NPT production run at 300 K. The temperature was controlled with a modified Berendsen thermostat (Bussi *et al.*, 2007), and the pressure with an isotropic Parrinello-Rahman, at 100 kPa. The iteration time step was set to 2 fs, with the Verlet integrator and LINCS (Hess, 2008) constraint. We used periodic boundary conditions. Configurations were sampled every 10 ps. All the simulations and their analyses were run as implemented in the GROMACS package (Pronk *et al.*, 2013).

### Immunofluorescence staining of patient skin

Skin samples (3 mm) were taken using a disposable punch after intradermal injection of lidocaine from the leg and forearm of Patient 1, and from leg, thigh and fingertip of Patient 3, and of a 5-year-old male with a suspected indifference to pain as control. Specimens were fixed overnight in Zamboni solution (American Mastertech), cryoprotected in 20% sucrose in PBS and sent in a refrigerated package to the laboratory of Telese. The skin samples were cut into 50-μm thick sections using a freezing slide microtome (Leica 2000R). Free-floating sections were processed for indirect immunofluorescence using antibodies to stain nerve fibres, myelin and vascular structures. To visualize nodal and paranodal architecture, rabbit antibodies against panNeurofascin (1:2000, courtesy of Prof P. Brophy), Nfasc186 (1:800, Cell Signaling Technology) or CASPR1 (1:1000, Abcam) were used. Axon and myelin were visualized with primary mouse antibodies against protein gene product 9.5 (PGP; AbD Serotec; 1:800), myelin basic protein (MBP; Santa Cruz Biotechnology; 1:800), or collagen IV (COLIV; Chemicon; 1:800) or a rabbit antibody against PGP (Biogenesis; 1:400). Species-specific secondary antibodies coupled with cyanine 2 and cyanine 3 fluorophores were used to visualize the structures of interest. ULEX Europaeus agglutinin 1 coupled with cyanine 5 was used to visualize blood vessels and epidermis. Skin sections were mounted on coverslips with agar, dehydrated in alcohol, clarified in methylsalicylate and finally mounted in DPX. Digital images were acquired using a non-laser confocal microscope (Apotome; Zeiss).

### Co-expression network analysis

Co-expression network analysis was used to investigate the function of *NFASC* across the human central and peripheral nervous system. This analysis was performed by using GTEx V6 gene expression data (Carithers *et al.*, 2015) to generate co-expression networks for each of the 13 brain tissues and tibial nerve RNA-seq data included within the GTEx study. The raw FPKM (fragments per kilobase of transcript per million mapped reads) values were corrected for known batch effects, age at death, sex and post-mortem interval, as well as unknown effects. The unknown effects were detected with the surrogate variable analysis (SVA) R Package (Leek and Storey, 2007) and correction was performed using ComBat (Johnson *et al.*, 2007). The resulting residuals were used to create a signed network using the blockwiseConsensusModules R function from the WGCNA R package (Langfelder and Horvath, 2008) for each of the 14 tissues. Next, the modules obtained in each of the 14 networks were assigned to cell types using the userListEnrichment R function implemented in the WGCNA R package, which measures enrichment between module-assigned genes and defined brain-related lists using a hypergeometric test. The same approach was used to annotate modules with Gene Ontology, REACTOME (Fabregat *et al.*, 2018) and KEGG (Kanehisa *et al.*, 2016) terms.

### Data availability

The data that support the findings of this study are available from the corresponding author, upon reasonable request.

## Results

### Identification of the *NFASC* variants

**Figure 2:**
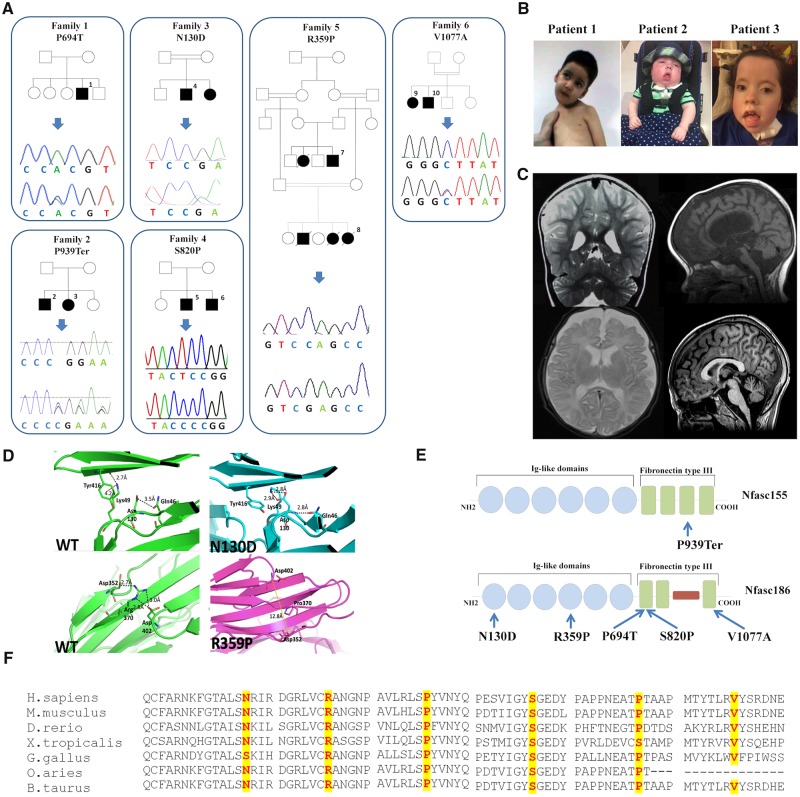
**Pedigrees, Sanger sequencing, multiple-sequence alignment of NFASC and clinico-radiological natural history of patients.** (**A**) Pedigrees of the six families carrying biallelic *NFASC* mutations and Sanger sequencing electropherograms confirming the mutations. (**B**) Facial pictures of Patients 1–3; note the facial hypomimia present in all cases, the muscle weakness and inability to hold his neck in Patient 1 carrying the p.P694T variant, and the tongue protrusion in Patients 2 and 3 carrying the frameshift mutation p.P939Ter. (**C**) MRIs of Patients 1–3 and 10 showing loss of cerebral white matter and atrophic changes of corpus callosum and brainstem: T_2_-weighted coronal MRI showing enlarged lateral ventricles in Patient 1 indicative of cortical volume loss (*top left*). T_1_-weighted sagittal MRI-sequence of Proband 2 displaying thin corpus callosum as well as cortical volume loss (*top right*). Axial T_2_-weighted sequence in Patient 3 showing progressive loss of cerebral white and grey matter (*bottom left*). T_1_-weighted sagittal MRI-sequence of Proband 10 displaying thin corpus callosum as well as cortical volume loss (*bottom right*). (**D**) *In silico* modelling of the 3D structure of the p.N130D and p.R359P variants. In the *top left* image [wild-type (WT) protein], a hydrogen bonding interaction with the side chain of glutamine 46 is visible. The residue of lysine 49 is distant from the point of mutation and forms a salt bridge with the carbonyl moiety of residue tyrosine 416. In addition, a weaker hydrophobic (cation-π) interaction forms between the side chains of lysine 49 and tyrosine 416 (red dashes, connecting the charged nitrogen atom with the centre of the phenyl ring). The *top right* image shows the different hydrogen bonding network formed in the mutant: the mutated aspartate residue forms three hydrogen bonding interactions, two involving its side chain and the side chain of residue lysine 49, and the third between its amino moiety and the side chain of residue glutamine 46. No interactions are visible between residues lysine 46 and tyrosine 416 that are further apart (∼6 Å) and with an unsuitable geometry. In the *bottom left *image (wild-type protein), a strong hydrogen bonding network connects residues aspartate 352, arginine 370 and aspartate 402 positioned on three different β-strands. The absence of the arginine residue in the mutant (*bottom right*) impedes the formation of the hydrogen bonding network, distancing the three β-strands. (**E**) A schematic representation of Nfasc155 and Nfasc186 showing the position of all *NFASC* variants. (**F**) Inter-species alignment performed with Clustal Omega shows the complete conservation down to invertebrates of the amino acid residues affected by the substitutions.

Biallelic *NFASC* variants were independently identified as the primary candidate genetic cause for the phenotype observed in the six families described herein (Fig. 2 and Table 1). WES carried out in Family 1 (Fig. 2) revealed a homozygous variant in *NFASC* (chr1:204948591C>A). This variant is predicted to cause a non-synonymous substitution in both the two major *NFASC* transcripts: NM_001160331.1: c.2113C>A (p.P705T) and NM_001005388.2: c.2080C>A (p.P694T). NM_001005388.2 is the canonical *NFASC *transcript encoding the longest protein isoform of 1241 amino acids corresponding to Nfasc186, while NM_001160331.1 encodes the protein isoform of 1165 amino acids corresponding to Nfasc155. Sanger sequencing-based segregation analysis was performed in Family 1 confirming the variant. Through GeneMatcher and SYNAPS Study Group, we identified seven additional cases, with clinical phenotypes partially overlapping that of Family 1. WGS was carried out in Proband 2 (Fig. 2A) at Genedx, Gaithersburg, USA, by massively parallel (NextGen) sequencing on an Illumina sequencing system with 100 bp or greater paired-end reads. This revealed a homozygous single base deletion (chr1:204986105delC) predicted to cause a frameshift variant in the Nfasc155 isoform NM_001160331.1: c.2816delC (p.P939Ter) and in *NFASC* transcript 3 NM_015090.3: c.2771delC (p.P924Ter). The frameshift variant is expected to cause loss of normal protein function either through protein truncation or nonsense-mediated mRNA decay. WES performed for one individual of Family 3 (Fig. 2A) detected a homozygous variant (chr1:204923488A>G), resulting in a missense change in both major transcripts: NM_001005388.2: c.388A>G (p.N130D) and NM_001160 331.1: c.298A>G (p.N124D), located within a 10 Mbp stretch of homozygosity. WES in the proband of Family 4 (Fig. 2A) revealed a homozygous variant (chr1:204951136 T>C) resulting in a missense change in both NM_0010053 88.2: c.2458T>C (p.S820P) and NM_001160331.1:c. 2491T>C (p.S831P). The index case of Family 5 (Fig. 2A) was subjected to WES as described elsewhere (Anazi *et al.*, 2017). A homozygous missense variant in *NFASC* was identified in chr1:204939816G>C. This variant is predicted in both major transcripts as a non-synonymous substitution: NM_001160331.1: c.1109G>C (p.R370P) and NM_00100 5388.2: c.1076G>C (p.R359P) and it segregates with the disease in the family (Patients 7 and 8). Family 6 was identified from replication cohort screening, where WES carried out in Probands 9 and 10 and their parents revealed a homozygous missense variant in chr1:204971817T>C. This variant is predicted to be a non-synonymous substitution only in the NF186 transcript: *NFASC* (NM_001005388.2:c.3230 T>C (p.V1077A) and it segregates with the disease in the family.

**Table 1:** *NFASC *intragenic variants identified in our cohort

Family	Genomic coordinates (GRCh37/hg19)	Variant	dbSNP 138	1000G	ESP6500	ExAC	gnomAD	SIFT	PolyPhen	Condel	CADD_ PHRED	GERP++
1	1:204948591–204948591	ENST00000339876.6	-	-	-	-	-	Deleterious (0)	Probably damaging (0.998)	Deleterious (0.919)	25.4	5.24
		Nfasc186:c.2080C>A:p.P694T										
		Nfasc155:c.2113C>A:p.P705T										
2	1:204986105–204986106	ENST00000430393.5	-	-	-	-	-	-	-	-	-	-
		Nfasc155:c.2816delC:p.P939Ter										
		Nfasc3:c.2771delC:p.P924Ter										
3	1:204923488–204923488	ENST00000339876.6	-	-	-	-	-	Tolerated (0.07)	Possibly damaging (0.67)	Deleterious (0.556)	21.5	5.37
		Nfasc186:c.388A>G:p.N130D										
		Nfasc155:c.298A>G:p.N124D										
4	1:204951136–204951136	ENST00000339876.6	-	-	-	-	-	Deleterious (0)	Probably damaging (1)	Deleterious (0.945)	28.6	5.55
		Nfasc186:c.2458T>C:p.S820P										
		Nfasc155:c.2491T>C:p.S831P										
5	1:204939816–204939816	ENST00000339876.6	-	-	-	-	-	-	Probably damaging (0.812)	Deleterious (0.75)	34	5.64
		Nfasc186:c.1076G>C:p.R359P										
		Nfasc155:c.1109G>C:p.R370P										
6	1:204971817–204971817	ENST00000339876.6	-	-	-	-	-	Tolerated (0.09)	Probably damaging (0.986)	Deleterious (0.73)	22.7	5.63
		Nfasc186:c.3230T>C:p.V1077A										

A CADD score ≥ 20 indicates that the variant is predicted to be the among the 1% most deleterious substitutions in the protein-coding parts of the human genome. A GERP++ score of close to 6 indicates a high evolutionary conservation of the NFASC sequence across species.

### Presentation of the neurofascin disease

Table 2 summarizes the core phenotypic features of 10 individuals (aged between 3 months and 21 years), including two patients from a Saudi Arabian family (Patients 7 and 8) described previously (Anazi *et al.*, 2017). Figure 2B and C illustrates the clinical features and MRI scans of affected individuals from each family. In all patients, psychomotor development was severely delayed (except Patients 5 and 6) in all domains. Affected individuals did not communicate, and none achieved purposeful hand movements or independent ambulation. Hypotonia was present from neonatal age in all cases. Impaired social interaction and only brief and occasional visual contact were noticed in all affected individuals by the first year of age. Several patients developed restricted patterns of interests and repetitive behaviours and frequently exhibited hand or head stereotypies. Moderate to severe intellectual disability was documented in all cases. None of the affected individuals attained intelligible speech. Because of muscle weakness and absent reflexes, individuals from Families 1, 2, 4 and 6 underwent detailed neurophysiological investigations as part of their diagnostic work-up and these showed a severe reduction in peripheral nerve conduction velocities in all cases. Patients from Families 1 and 2 exhibited the most profound phenotype with severe demyelinating and axonal neuropathy at neurophysiological investigations. Non-specific EEG abnormalities including slow background activities were observed in several families although affected individuals had no history of seizures. Given that *NFASC* is expressed in various brain regions, with the highest transcript level in the white matter region (Supplementary Fig. 7), MRI studies of Patients 1–3, 7, 9 and 10 showed loss of cerebral white matter, which was more severe in some affected individuals (Patients 1–3) and also associated with atrophic changes mainly involving the corpus callosum and the brainstem (Fig. 2C). T_2_-weighted coronal MRI showing enlarged lateral ventricles in Patient 1 indicative of cortical volume loss (Fig. 2C). Interestingly, similar MRI patterns are also presented in *CASPR1*-deficient patients with brainstem and corpus callosum atrophy (Hengel *et al.*, 2017). Previously, cerebellar atrophy has been directly associated with loss of *NFASC* in both Purkinje and basket neurons causing abnormal basket axon collateral branching and targeting to the axon initial segment, leading to extensive pinceau disorganization, Purkinje neuron degeneration and severe ataxia (Buttermore *et al.*, 2012). Because of their severe muscle weakness, the majority of affected individuals had non-expressive/hypomimic face, and several craniofacial dysmorphisms including bi-temporal narrowing, high and wide nasal bridge, micrognathia, glossoptosis and highly arched palate (Fig. 2B). The two American children had more severe phenotype and also developed profound sensorineural hearing loss, feeding difficulties requiring gastrostomy and respiratory difficulties. The younger of the two Saudi Arabian children exhibited skeletal chest deformities and generalized hypertonia (mainly affecting upper limbs) and died at the age of 4 months, presumably due to aspiration defects. The two French children identified from replication cohort screening have an older age of onset compared to the rest of the patients, and the fact that the Nfasc155 transcript is intact while the missense variant is only present in Nfasc186 could imply a different mode of disease evolution.

**Table 2:** Genetic, clinical and neurophysiology details of patients with *NFASC* variants

**Patient**	**1**	**2**	**3**	**4**	**5**	**6**	**7**	**8**	**9**	**10**	**Smigiel *et al.* (2018)**	**Monfrini *et al.* (2019)**	**Monfrini *et al.* (2019)**
Gender	Male	Male	Female	Male	Male	Male	Male	Female	Female	Male	Female	Female	Male
Origin	Moroccan	American	American	Arab Iraqi	Israeli	Israeli	Saudi Arabian	Saudi Arabian	Algerian	Algerian	Polish	Italian	Italian
Current age	4 years	1 year and 6 months	10 years	3 years	12 years	16 years	2 years 6 months	4 months (at time of death)	21 years	16 years	1 year	22 years	15 years
Variant	c.2080C>A; p.P694T	c.2816delC; p.P939Ter	c.2816delC; p.P939Ter	c.388A>G; p.N130D	c.2458T>C; p.S820P	c.2458T>C; p.S820P	c.1076G>C; p.R359P	c.1076G>C; p.R359P	c.3230T>C: p.V1077A	c.3230T>C: p.V1077A	c.2491C>T; p.R831Ter	c.3365T>A; p.V1122E	c.3365T>A; p.V1122E
Age at onset of initial symptoms	Neonatal hypotonia	Neonatal hypotonia	Neonatal hypotonia	Neonatal hypotonia	Hypotonia during the first 6 months of life	Hypotonia during the first 2 months of life	Hypotonia during first 3 months of life	Hypotonia during first 3 months of life	Neonatal hypotonia	Hypotonia/ataxia started at 15–16 months	Congenital hypotonia, amimia, areflexia	Spastic limb hyperotnia, hyperreflexia	Episodes of aggression, intense anxiety
Developmental delay	+	+	+	+	+	+	+	+	+	+	+	+	+
Intellectual disability	+	+	+	+	+	+	+	+	+	+	_	_	+
Speech impairment	+	+	+	+	+	+	+	+	+	+	NA	+	+
Hypotonia / weakness	+	+	+	+	+	+	+	+	+	+	+	+	+
Neurological examination	Weakness, absent reflex, abnormal sensation, no walk	Weakness, absent reflex, no walk	Weakness, no walk	Weakness, no walk	Weakness, no walk	Weakness, no walk	Weakness, no walk	Weakness, no walk	No walk	Walk with a walking frame	Contractures of fingers and toes, no reaction to touch or pain	Psychomotor delay, cerebellar syndrome, hyperreflexia, spasticity, dysphagia, myoclonic jerks	Psychomotor delay, cerebellar syndrome, anxiety, aggression
Sensory NCS	Reduced CV, normal Amp	NT	NT	NT	SSR in hand and food	NT	Normal	NT	Normal CV, reduced Amp in ulnar nerve	Normal	reduced CV	Reduced CV (except normal CV and reduced Amp median nerve)	Marginally reduced CV
Motor NCS	Reduced CV (except normal CV ulnar nerve), normal Amp (except peroneal nerve reduced)	NT	Severely reduced CV, normal Amp (except peroneal nerve reduced)	NT	NT	NT	Normal	NT	Normal	Normal	NT	Reduced CV (except normal CV ulnar nerve)	Reduced CV
EMG	NT	NT	Acute and chronic denervation, PSW	NT	NT	NT	Normal	NT	Chronic denervation- reinnervation	Chronic denervation- reinnervation	NT	NT	NT
MRI	Atrophic changes and white matter loss	Cerebral white matter loss	Cerebral white matter loss. Severe atrophy of corpus callosum	NT	Normal	Normal	Diffuse white matter T_2_ hyperintensity	NT	Cerebellar atrophy	Cerebellar atrophy	Ischaemic and hypoxic changes	Mild cerebellar atrophy, mild diffuse white matter T_2 _hyperintensity	Mild diffuse white matter T_2_ hyperintensity

− = absent; + = present; Amp = amplitude; CV = conduction velocity/velocities; NA = not applicable; NCS = nerve conduction studies; NT = not tested; PSW = positive sharp waves; SSR = sympathetic skin response.

### Molecular modelling

To examine if these disease-associated variants affect *NFASC* structure and paranodal junction stability, 500 ns molecular dynamics simulations were performed using mutant ectodomain models based on the humanized wild-type as a template. Rearrangements on the variant sites showed that even though p.R370P gives no significant fluctuations in terms of protein conformation and movement, a weaker hydrophobic bond in residues 49 and 416 of the p.N130D protein can make its conformation less regular (Fig. 2). While the wild-type and p.R370P settle in a stationary state, p.N130D results in a slightly compressed mutant. This is evident in their backbone root mean squared deviation (RMSD) (Supplementary Fig. 4), which measures the divergence of the mutant protein structure from its initial structure over the course of the simulation and radius of gyration (Rg), which gives an indication on the change in size of the protein. In all cases, the most mobile portion of the chain is residue 237 (corresponding to residue 248 in the transcript containing the p.R370P variant) as seen (Supplementary Fig. 4) in their root mean squared fluctuation (RMSF), which indicated how much each residue diverges form its initial position. This arginine residue is exposed to the aqueous solvent and part of a larger loop, probably flexible and crucial to protein dynamics. The RMSF further indicates that in all cases on average the overall mobility of the backbone is not particularly affected by variants.

### Surface expression and protein levels of *NFASC* variants

**Figure 3:**
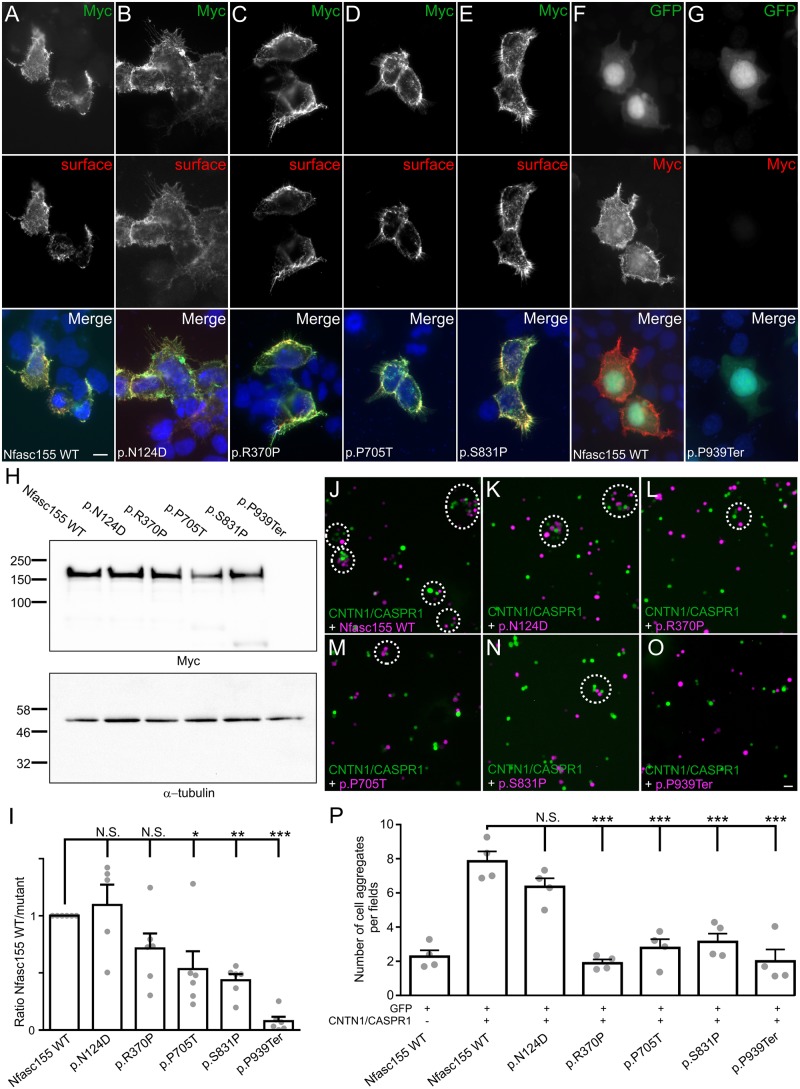
**Membrane targeting of Nfasc155 variants. **The homozygous Nfasc155 variants associated with a severe pathology diminish Nfasc155 protein level. The variants p.P705T and p.P939Ter inhibit the association of CASPR1/CNTN1 with Nfasc155. (**A**–**E**) HEK cells were transfected with Myc-tagged Nfasc155 variants and surface Nfasc155 was monitored by incubating the live cells with anti-Nfasc155 IgG (red) prior to fixation and permeabilization. Nfasc155 was then revealed using an anti-Myc antibody (green). Wild-type Nfasc155, but also the variants p.N124D, p.R370P, p.P705T and p.S831P were readily targeted at the cell surface, and did not show signs of intracellular retention. (**F** and **G**) HEK cells were co-transfected with GFP (green) and either wild-type Nfasc155 or p.P939Ter variant, then cells were immunostained for Myc (red). In contrast to wild-type Nfasc155, p.P939Ter was not detectable, indicating that this mutation strongly affects Nfasc155 expression. Nuclei are stained DAPI (blue). Scale bar = 10 µm. (**H**) Western blot analysis of HEK cells transfected with Nfasc155 variants and revealed with anti-Myc antibodies or anti-a-tubulin antibodies as loading control. (**I**) Protein expression levels were analysed by normalizing the signals to the corresponding α-tubulin signal, then to wild-type Nfasc155 in four independent experiments. The expression levels of p.P705T, p.S831P and p.P939Ter variants were significantly decreased compared to wild-type Nfasc155 (Mann-Whitney test). *P < 0.01; **P < 0.005; ***P < 0.001; by unpaired two-tailed Student’s *t*-tests for two samples of equal variance and by one-way ANOVA followed by Bonferroni’s *post hoc* tests. Bars represent mean and SEM. Molecular weight markers are shown on the *left* (in kDa). N.S. = not significant. (**J**–**O**) N2A cells were transfected with GFP (green) in combination with CNTN1 and CASPR1 and were incubated for 2 h with cells transfected with tandem tomato (magenta) and wild-type Nfasc155 (**J**), p.P705T (**M**) or pP939Ter (**O**). As negative control, N2A cells transfected with Nfasc155 (magenta) were incubated with cells transfected with GFP. (**P**) The numbers of green and magenta cell aggregates (dashed line circles in **J**–**O**) per visualization field were quantified in each condition (*n* = 4 experiments for each condition). p.R370P, p.P705T, p.S831P and p.P939Ter mutations significantly decreased aggregate formation. ***P < 0.001 by unpaired two-tailed Student’s *t*-tests and by one-way ANOVA followed by Bonferroni’s *post hoc* tests. Bars represent mean and SEM. Scale bar = 20 µm.

To determine the impact of genetic variants on *NFASC* function, mutations were introduced in plasmids encoding Myc-tagged human Nfasc155 or Nfasc186. In the case of the p.P939Ter variant, the Myc-tag was inserted before the premature stop codon in the aberrant C-terminal extension. HEK cells were then transfected with these constructs and the neurofascin cell surface expression was monitored by immunocytochemistry (Fig. 3 and Supplementary Fig. 1). To detect surface expression of *NFASC* isoforms, living transfected cells were incubated with antibodies to Nfasc155 or Nfasc186 for 20 min, then cells were fixed, permeabilized and immunostained for Myc. Wild-type Nfasc155 (Fig. 3A) and Nfasc186 (Supplementary Fig. 1 and 2) are readily expressed at the cell surface and a complete co-localization is observed between the cell surface proteins and the Myc-labelled proteins after permeabilization. Most of the *NFASC* variants did not seem to affect the surface expression of Nfasc155 or Nfasc186, and a clear co-localization could be detected between surface-labelled and Myc-labelled proteins (Fig. 3B–E and Supplementary Fig. 1). Only the p.P939Ter variant strikingly affected the expression of Nfasc155. This mutant protein was not detectable at the cell surface, or after Myc labelling. To confirm this observation, cells were co-transfected with GFP then fixed and stained for Myc. When cells were co-transfected with wild-type Nfasc155, all GFP-labelled cells also co-expressed Myc-tagged Nfasc155 at the cell surface (Fig. 3F). By contrast, no cell surface immunostaining was detected in cells co-transfected with p.P939Ter (Fig. 3G). This suggested that the variant abolished Nfasc155 expression.

To confirm this observation further, we examined the protein levels of Nfasc155 variants by western immunoblotting (Fig. 3H). The protein levels of p.P705T, p.S807P and p.P939Ter mutants were found importantly decreased compared to wild-type Nfasc155 (Fig. 3I). Particularly, p.P939Ter was not detectable on immunoblots even at lower molecular weight. By contrast, the p.N124D and p.R370P variants had no significant effect on Nfasc155 protein levels. Because p.P939Ter is a frameshift variant inducing a premature stop codon before the transmembrane domain of Nfasc155, we suspected that this protein may be released in the culture medium. We thus harvested the cell culture medium and immunoblotted the released fraction for Myc antibodies. No signal was detected in the released fraction even after acetone precipitation and concentration of the sample (data not shown), indicating that the p.P939Ter variant abolishes Nfasc155 expression.

### Nfasc155 variants inhibit the interaction with CNTN1/CASPR1

Nfasc155 is known to interact with CASPR1 and CNTN1 at the paranodal regions of myelinated axons, and deletion of *CNTN1* or *CASPR1* leads to important conduction slowing in myelin-deficient mice (Sherman *et al.*, 2005). Because the affected patients presented demyelinating features, we suspected that Nfasc155 variants may alter its interaction with these axonal partners. To test this hypothesis, N2A cells were co-transfected with CNTN1, CASPR1, and GFP, then incubated for 2 h with cells co-expressing Nfasc155 and tandem tomato (Fig. 3). The number of cell aggregates was then quantified. As negative control, N2A cells expressing Nfasc155 were incubated with cells expressing GFP alone (Fig. 3). In keeping with a previous report (Labasque *et al.*, 2014), we found that N2A cells expressing *CNTN1/CASPR1* readily interact and form aggregates with Nfasc155-expressing cells (Fig. 3J–O). By contrast, only a minimal interaction was observed between Nfasc155-expressing cells and GFP alone (Fig. 3P). Both p.P705T and p.P939Ter variants abolished the interaction between Nfasc155 and CNTN1/CASPR1, indicating that these variants impact Nfasc155 function (Fig. 3L, O and P). To determine whether the genetic variants may also affect the surface expression of Nfasc186 or the interaction of Nfasc186 with gliomedin, we tested these on a western blot and also tested the binding of gliomedin-Fc on Nfasc186-transfected cells. The blot showed p.N130D had a higher expression level, while the other mutants had a normal expression level compared to wild-type Nfasc186 (Supplementary Fig. 2). In addition, no alterations on gliomedin binding were observed on any of the Nfasc186 variants (Supplementary Fig. 1).

### Immunofluorescence staining analysis

**Figure 4:**
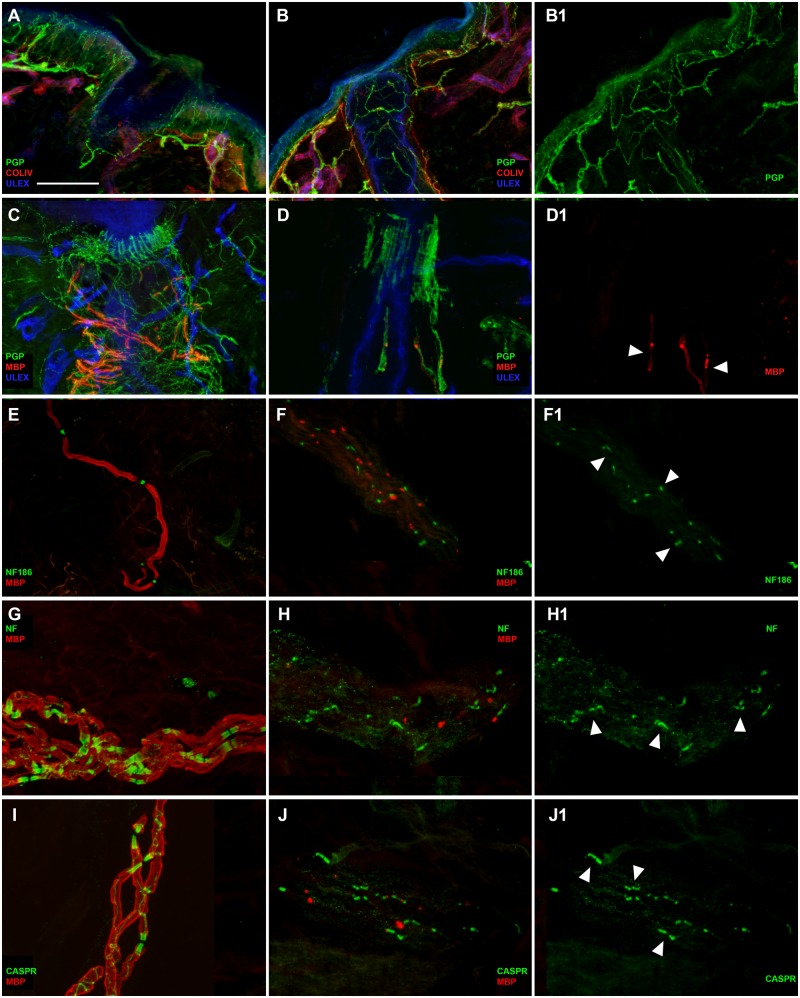
**Severe involvement of peripheral myelinated fibres and partial disruption of Nfasc155 at the paranode in Patient 1. **Confocal images of cutaneous innervation from hairy skin (leg and arm) of the patient compared to a control showing well preserved unmyelinated fibres in the epidermis and dermis (**B** and **B1** compared to **A**), severe loss of myelinated fibres around a hair follicle and the few remaining ones presenting a very weak MBP (red) immunoreactivity (**D**, **D1** arrowheads compared to **C**), in a nerve bundle with few remaining myelinated fibres presenting a very faint staining with MBP antibody presence of nodes marked with Nfasc186 (green) (**F** and **F1** compared to **E**), paranodes marked with panNeurofascin (green) (**H**, **H1** arrowheads compared to **G**) and paranodes marked with CASPR (green) (**J** and **J1** arrowheads compared to **I**). Scale bar = 100 µm in **A**, **B**, **B1**; 50 µm in **C**, **D**, **D1**; 30 µm in **F**–**J1**.

In protein gene product 9.5/collagen IV (PGP/COLIV) double-stained sections of both arm and leg samples from Patient 1 (Fig. 4), cutaneous innervation appeared quite preserved (Fig. 4B and B1 compared to A). In particular, epidermal nerve fibres as well as sudomotor and pilomotor nerve fibres appeared normally represented and regularly distributed. Conversely, a severe loss of myelinated fibres was observed around the hair follicle as evidenced by the simplification of the so called ‘palisade-like’ endings originating from the few remaining myelinated fibres (Fig. 4D and D1 compared to C). Myelin staining appeared very weak with some bright spots along the nerve course (Fig. 4D and D1). No further myelinated fibres were observed in MBP/PGP double-stained sections. However, small nerve fascicles were recognized in the deep dermis in sections double-stained with Nfasc186/MBP (Fig. 4F and F1 compared to E), panNeurofascin/MBP (Fig. 4H and H1 compared to G) and *CASPR1*/MBP (Fig. 4J and J1 compared to I). Also, in these fascicles the MBP-immunoreactivity appeared very weak or absent and again bright spots were present along the nerve course. Nfasc186 immunoreactivity was normally expressed in the nodal region along few profiles of nerve fibres, as well as panNeurofascin and *CASPR1* immunoreactivity at the paranodes. In general, we observed a severe loss of myelinated fibres with a preservation of the unmyelinated ones. The abnormal immunoreactivity to MBP antibody suggests a severe myelin involvement leading to probable nerve degeneration. In contrast, the expression of Nfasc186 in the nodal regions and of panNeurofascin and *CASPR1* in the paranodal regions appeared normal, although limited to the few remaining fibres. The observation of few paranodes stained with *CASPR1* as well as with panNeurofascin suggests that the absence of Nfasc155 is partial in Patient 1.

**Figure 5:**
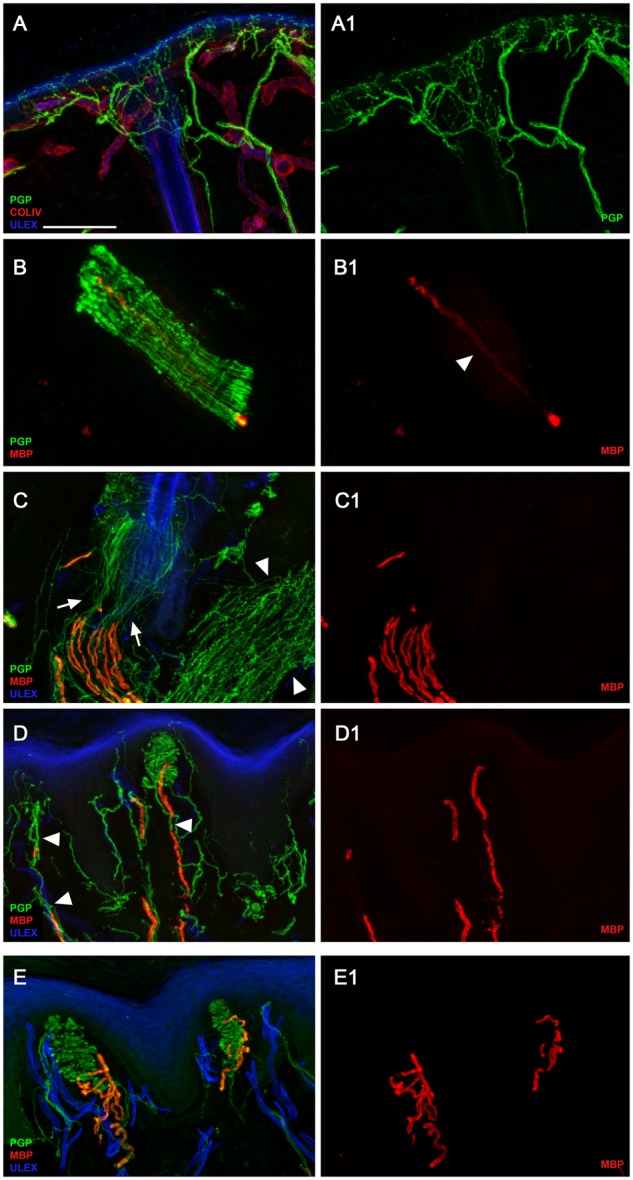
**Severe involvement of peripheral myelinated axons in Patient 3 with complete lack of Nfasc155 expression at paranodes. **Confocal images of cutaneous innervation from hairy and glabrous skin (thigh and fingertip) of the patient compared to a control showing quite preserved unmyelinated fibres in the epidermis (**A** and **A1**) and around autonomic annexes (see arrector pili muscle, arrowheads in **C**); severe loss of myelinated fibres with evidence in a nerve fascicle of few segments with severe myelin abnormalities in the distal site (leg) (**B** and **B1**); more preserved myelinated fibres with evidence around a hair follicle of several myelinated fibres showing loss of myelin in their distal segments in the more proximal site (thigh) (**C**, arrowheads in **C1**); loss of mechanoreceptors and myelinated fibres (compared to the control in **E** and **E1**) and morphological abnormalities of myelinated fibres with tracts of demyelination in the fingertip (**D**, arrowheads in **D1**) (compared to the control in **E** and **E1**). Scale bar = 100 µm in **A**, **A1**, **C**, **C1**, **D**, **D1**, **E**, **E1** and 30 µm in **B** and **B1**.

In PGP/ColIV double-stained sections from Patient 3 (Fig. 5), cutaneous innervation appeared quite preserved in the epidermis (Fig. 5A and A1) and around sweat glands and other autonomic annexes (Fig. 5C) in all three examined sites. Observing MBP/PGP double-stained sections from distal leg, it was evident a severe loss of myelinated fibres surrounding the hair follicle with few segments showing severe myelin abnormalities (Fig. 5B, B1). In MBP/PGP double stained skin sections from the proximal site (thigh), myelinated fibres appeared more preserved with several myelinated segments visible around a hair follicle (Fig. 5C and C1). Similarly, we could sample a definitely larger amount of myelinated fibres from the fingertip (Fig. 5D and D1) compared to the other two sites, although there was an evident loss of mechanoreceptors and myelinated fibres compared to a control case (Fig. 5E and E1). At higher magnification, we observed abnormalities of myelin sheet and enlargement of the nodal gap (Supplementary Fig. 3A). In Nfasc186/MBP images, we observed a normal expression of neurofilament staining in the node (Supplementary Fig. 3B). The nodal structure appeared identical by visualizing the node with the anti-panNeurofascin or the anti-Nfasc186 antibodies (Supplementary Fig. 3C). *CASPR1* immunoreactivity was completely absent in the paranodal regions (Supplementary Fig. 3D). Additionally, an apparently normal distribution of voltage-gated sodium (Na_v_) channels appeared in MBP/Na_v_ double-staining images in several nodes that appeared larger, however less compacted than in the control skin (Supplementary Fig. 3F). Moreover, a granular distribution of Na_v_ was evident along the axonal profiles of large fibres devoid of myelin (arrows in Supplementary Fig. 3E) that suggested a Na_v_ remodelling after demyelinating phenomena. Those data indicate that the frameshift variant in Patient 3, which induces a complete lack of Nfasc155 expression, does not provoke a complete disruption of the node. There was, however, a severe alteration of the paranode as showed by the complete absence of the *CASPR1*, the physiological ligand of Nfasc155, and a severe effect on myelination and/or maintenance of myelin and survival of large fibres, which appears to be length-dependent. Interestingly, small fibres, both somatic and autonomic appeared less affected by the variant in both patients.

### *NFASC* cell-specific expression and co-expression analysis

We first explored the expression of *NFASC* in the central and peripheral nervous system using publicly available cell-specific transcriptomic data (Zhang *et al.*, 2016; Zeisel *et al.*, 2018). In keeping with the known function of *NFASC*, RNA sequencing of purified cell types derived from human cortex demonstrated that *NFASC* was most highly expressed in oligodendrocytes though there was also evidence of expression in neurons and, to a lesser extent, astrocytes and endothelial cells (Supplementary Fig. 6). We also found that *NFASC* expression was highest in newly formed oligodendrocytes rather than myelin-forming or mature oligodendrocytes. Next, we investigated the function of *NFASC* using gene co-expression analysis to construct networks for all 13 human brain regions sampled by the Genotype Tissue Expression Consortium (Supplementary Table 2) (Human Genomics, 2015). In the anterior cingulate cortex, *NFASC* was located with high confidence (module membership = 0.74) within a co-expression module, which is enriched for genes involved in synaptic transmission (GO:0099536, chemical synaptic transmission FDR-corrected *P*-value = 6.68 × 10^−10^) and expressed specifically in neurons (neuron module in cortex, FDR-corrected *P*-value = 6.0 × 10^−72^). Furthermore, this module was enriched for genes known to be associated with monogenic forms of intellectual disability (FDR-corrected *P*-value 2.12 × 10^−3^, Supplementary Fig. 5).

## Discussion

Homozygous variants in *NFASC* lead to a neurodevelopmental disorder that includes—in some families—a chronic demyelinating neuropathy. Radiological features in some *NFASC* individuals included central white matter loss and atrophy of the corpus callosum and the brainstem (Fig. 2C). As part of their phenotype, several affected individuals also exhibited features of central demyelination and clinical and neurophysiological evidences of chronic peripheral demyelinating neuropathy associated with severe reduction in nerve conduction velocities and prolonged motor latencies (Table 2 and Supplementary Table 1). Interestingly, these patients have a similar phenotype to patients with *CNTNAP1/*CASPR1 mutations; however, without any epilepsy, which has been reported in *CASPR1*-mutated patients. It is possible that a different role is played by *CASPR1* and *NFASC* in neuron excitability at CNS level (Laquerriere *et al.*, 2014; Hengel *et al.*, 2017).

Immunoglobulin G subclass 4 (IgG4) antibodies to Nfasc155 and Nfasc186 have been identified in 3–18% of patients with chronic inflammatory demyelinating polyradiculoneuropathy (Querol *et al.*, 2014; Ogata *et al.*, 2015; Delmont *et al.*, 2017). Nfasc155 IgG4 antibodies are associated with specific clinical features including an early onset of the CIDP disease, a distal predominant pattern of weakness, and the presence of sensory ataxia and tremor. A cerebellar origin of the tremor has been hypothesized in some cases (Devaux, 2014; Querol *et al.*, 2014; Ogata *et al.*, 2015). Also, and most importantly to the present study, up to 8% of CIDP patients with IgG4 Nfasc155 showed evidence of CNS demyelination (Devaux *et al.*, 2016) and anti-Nfasc155 antibodies were identified at high frequency in Japanese patients with combined central and peripheral demyelination (Kawamura *et al.*, 2013), although this was not replicated in the Caucasian population (Cortese *et al.*, 2016).

The majority of *NFASC *mutated individuals identified in this study were found with homozygous missense variants affecting highly conserved residues within the Ig-like domains or in the fibronectin type III domains of the neurofascin protein (Fig. 2E). One family (Family 2) was identified with a homozygous frameshift deletion predicted to result in a premature truncation of the protein. Among our patients, affected individuals from Family 2 carrying the p.P939Ter frameshift deletion exhibited the more severe phenotype with marked neurodevelopmental impairment and profound muscle weakness associated with severe reduction of conduction velocities on neurophysiological investigations. Given the history of consanguineous marriages in these families, other recessive variants may also have contributed to the clinical phenotype, as in Proband 8 from Family 5 who died prematurely, presumably because of aspiration due to respiratory difficulties.

The results from our functional analyses indicate abnormal Nfasc155 interaction with CNTN1 and CASPR1 as an important disease mechanism in the *NFASC*-related genetic disease. In addition, loss of Nfasc155 surface expression may also be implicated in the phenotype observed in the affected individuals. Immunofluorescence studies on Patients 1 and 3 detected a severe loss of myelinated fibres with a preservation of the unmyelinated ones, and while the expression of Nfasc186 in the nodal regions appeared normal in both patients, the absence of Nfasc155 in the paranodal region was only partial in Patient 1. Western blot showed that the protein levels of several Nfasc155 mutants including p.P705T and p.P939Ter were importantly decreased compared to wild-type Nfasc155. These biochemical findings support the morphological findings and indicate that there may be an incomplete derangement of the paranodal region in some patients due to a reduction of glial Nfasc155. This could explain the relatively milder phenotype of Patients 6 and 7 as well as that of the recently described patient with a similar neurodevelopmental syndrome (Smigiel *et al.*, 2018).

Of interest, in cell-specific *in silico* studies, NFASC is mostly expressed in newly formed oligodendrocytes, and found to be enriched for genes associated with monogenic forms of intellectual disability. Given recent evidence that the generation of new myelin segments is an important form of plasticity that can be used to modify the properties of circuits in the CNS (McKenzie *et al.*, 2014; Xiao *et al.*, 2016), these observations may be significant and provide some explanation for the neurodevelopmental features of individuals carrying biallelic *NFASC* variants. We could further speculate that in at least some brain regions *NFASC* may play an important role in synaptic transmission and that this potentially might explain how biallelic variants in the gene result in central neurological features.

Interestingly, homozygous *NFASC*-null mice are born with a normal appearance but die suddenly 7 days after birth when the postnatal transition to saltatory conduction is occurring in the CNS and PNS. Over-expression of either of the two neuronal isoforms (Nfasc186 or Nfasc140) helps them survive into adulthood but mice are ataxic, presumably because they do not have intact paranodal axoglial junctions in their myelinated nerves (Sherman *et al.*, 2005).

Recently, a homozygous splice-site variant in *NFASC* was identified in an individual with neurodevelopmental impairment, hypotonia and areflexia (Farwell Hagman *et al.*, 2017). Another family was found within a large genetic study carrying a homozygous *NFASC* variant associated spinal muscular atrophy (Smigiel *et al.*, 2018) and a homozygous missense mutation leads to significant loss of NFASC protein in induced pluripotent stem cell-derived neurons from affected subjects (Monfrini *et al.*, 2019).

Despite these reports, the implication of *NFASC* mutations in the development of human diseases remains to be fully elucidated and warrants further investigations on a larger scale. Our results strongly establish the association of biallelic *NFASC* variants in the complex central and peripheral nervous system involvement presented by the 10 affected individuals described in this study. The notion of a genetic *NFASC* disease further delineates an emerging spectrum of human neurological disorders caused by variants in cell adhesion and ankyrin-binding genes involved in neurite extension, axonal guidance, synaptogenesis, myelination and neuron-glial cell interactions.

## Supplementary Material

awz248_Supplementary_Data

## Abbreviation

WES: whole exome sequencing

## Appendix 1

### SYNAPS Study Group

Full details are available in the Supplementary material.

Stanislav Groppa, Blagovesta Marinova Karashova, Wolfgang Nachbauer, Sylvia Boesch, Larissa Arning, Dagmar Timmann, Bru Cormand, Belen Pérez-Dueñas, Jatinder S. Goraya,, Tipu Sultan, Jun Mine, Daniela Avdjieva, Hadil Kathom, Radka Tincheva, Selina Banu, Mercedes Pineda-Marfa, Pierangelo Veggiotti, Michel D. Ferrari, Arn M. J. M. van den Maagdenberg, Alberto Verrotti, Giangluigi Marseglia, Salvatore Savasta, Mayte García-Silva, Alfons Macaya Ruiz, Barbara Garavaglia, Eugenia Borgione, Simona Portaro, Benigno Monteagudo Sanchez, Richard Boles, Savvas Papacostas, Michail Vikelis, James Rothman, Dimitri Kullmann, Eleni Zamba Papanicolaou, Efthymios Dardiotis, Shazia Maqbool, Shahnaz Ibrahim, Salman Kirmani, Nuzhat Noureen Rana, Osama Atawneh, Shen-Yang Lim, Farooq Shaikh, George Koutsis, Marianthi Breza, Salvatore Mangano, Carmela Scuderi, Eugenia Borgione, Giovanna Morello, Tanya Stojkovic, Massimo Zollo, Gali Heimer, Yves A. Dauvilliers, Carlo Minetti, Issam Al-Khawaja, Fuad Al-Mutairi, Sherifa Hamed, Menelaos Pipis, Conceicao Bettencourt, Simon Rinaldi.
