# Supplementary material for: Biallelic mutations in neurofascin cause neurodevelopmental impairment and peripheral demyelination
Source: Brain. 2019 Sep 9;142(10):2948–64. doi: 10.1093/brain/awz248 (PMC6763744; doi:10.1093/brain/awz248)
Supplement: awz248_Supplementary_Data [file awz248_supplementary_data.zip › awz248-Suppl_data/Supplementary_Data2.pdf]

wellcometrust

SYNAPSE Study Group

A team of world-class researchers working towards a better understanding of the genetic basis in neurological disorders

Supported by the Wellcome Trust

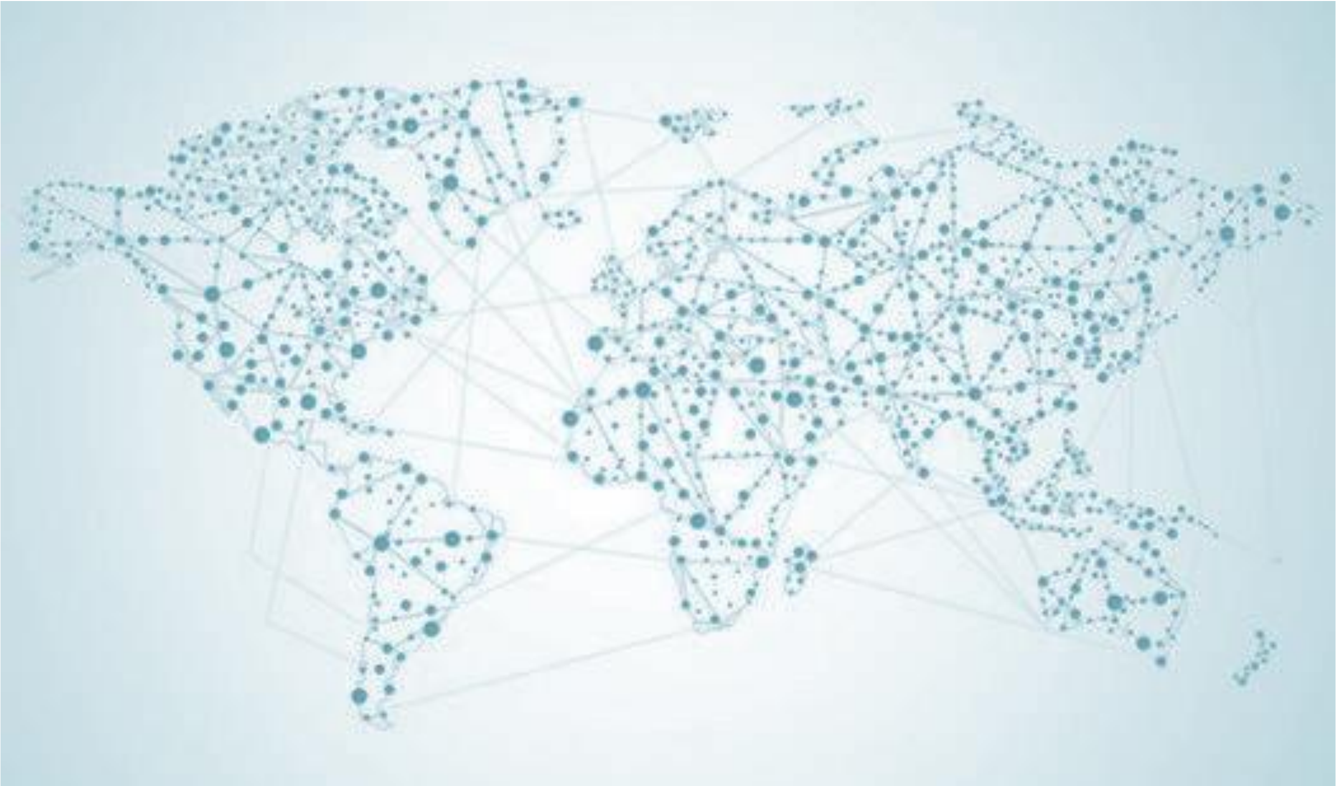

Collaborators

Prof Stanislav Groppa
Affiliation: Department of Neurology and Neurosurgery, Institute of Emergency Medicine, Chisinau, Republic of Moldova.
Email: sgroppa@gmail.com

Dr. Blagovesta Marinova Karashova
Affiliation: Department of Paediatrics, Medical University of Sofia, Sofia 1431, Bulgaria
Email: blagovestakarashova@gmail.com

Prof Lionel Van Maldergem
Affiliation: Centre of Human Genetics, University Hospital Liege, Liege 4000, Belgium
Email: lvanmaldergem@chu-besancon.fr

Dr. Wolfgang Nachbauer
Affiliation: Department of Neurology, Medical University Innsbruck, Anichstrasse 35, Innsbruck 6020, Austria
Email: Wolfgang.Nachbauer@i-med.ac.at

Prof. Sylvia Boesch
Affiliation: Department of Neurology, Medical University Innsbruck, Anichstrasse 35, Innsbruck 6020, Austria
Email: sylvia.boesch@i-med.ac.at

Dr. Larissa Arning
Affiliation: Department of Human Genetics, Ruhr-University Bochum, Bochum 44801, Germany
Email: Larissa.Arning@ruhr-uni-bochum.de

Prof. Dagmar Timmann
Affiliation: Braun Neurologische Universitätsklinik Universität Essen, Hufelandstr 55, Essen D-45122, Germany
Email: Dagmar.Timmann-Braun@uni-duisburg-essen.de

Prof. Bru Cormand
Affiliation: Department of Genetics, Universitat de Barcelona, Barcelona 08007, Spain
Email: bcormand@ub.edu

Dr. Belen Pérez-Dueñas
Affiliation: Hospital Sant Joan de Deu, Esplugues de Llobregat 08950, Barcelona, Spain
Email: bperez@sjdhospitalbarcelona.org

Dr Gabriella Di Rosa, MD, PhD
Affiliation: Department of Pediatrics, University of Messina, Messina 98123, Italy
Email: gdirosa@unime.it

Prof. Jatinder S. Goraya, MD, FRCP
Affiliation: Division of Paediatric Neurology, Dayanand Medical College & Hospital, Ludhiana, Punjab 141001, India
Email: gorayajs@gmail.com

Prof. Tipu Sultan
Affiliation: Division of Paediatric Neurology, Children's Hospital of Lahore, Lahore 381-D/2, Pakistan
Email: tipusultanmalik@hotmail.com

Prof Jun Mine
Affiliation: Department of Paediatrics, Shimane University, Faculty of Medicine, Izumo, 693-8501, Japan
Email: jmine@med.shimane-u.ac.jp

Prof. Daniela Avdjieva,
Affiliation: Department of Paediatrics, Medical University of Sofia, Sofia 1431, Bulgaria
Email: davdjieva@yahoo.com

Dr. Hadil Kathom,
Affiliation: Department of Pediatrics, Medical University of Sofia, Sofia 1431, Bulgaria
Email: hadilmk@gmail.com

Prof.Dr Radka Tincheva
Affiliation: Head of Department of Clinical Genetics, University Pediatric Hospital, Sofia 1431, Bulgaria
Email: radka.tincheva@gmail.com

Prof. Selina Banu
Affiliation: Neurosciences Unit, Institute of Child Health and Shishu Shastho Foundation Hospital, Mirpur, Dhaka 1216, Bangladesh
Email: selinabanu17@gmail.com

Prof. Mercedes Pineda-Marfa
Affiliation Servei de Neurologia Pediàtrica, l'Hospital Universitari Vall d'Hebron, Barcelona 08035, Spain
Email: pineda@hsjdbcn.org

Prof. Pierangelo Veggiotti
Affiliation: Unit of Infantile Neuropsychiatry Fondazione, Istituto Neurologico "C. Mondino" IRCCS, Via Mondino 2, Pavia 27100, Italy
Email: pierangelo.veggiotti@unipv.it

Prof. Michel D. Ferrari
Affiliation: Leiden University Medical Center, Albinusdreef 2, Leiden 2333, Netherlands
Email: M.D.Ferrari@lumc.nl

Prof Arn M J M van den Maagdenberg
Affiliation: Leiden University Medical Center, Albinusdreef 2, Leiden 2333, Netherlands
A.M.J.M.van_den_Maagdenberg@lumc.nl

Prof. Alberto Verrotti
Affiliation: University of L'Aquila, L'Aquila, Italy
Email: verrottidipianella@univaq.it

Prof Giangluigi Marseglia
Affiliation: Department of Pediatrics, University of Pavia, IRCCS Policlinico "San Matteo", Pavia 27100, Italy

Email: gl.marseglia@smatteo.pv.it

Dr. Salvatore Savasta
Affiliation: Division of Pediatric Neurology, Department of Pediatrics, University of Pavia, IRCCS Policlinico "San Matteo", Pavia 27100, Italy
Email: S.Savasta@smatteo.pv.it

Dr. Mayte García-Silva
Affiliation: Hospital Universitario 12 de Octubre, Madrid 28041, Spain
Email: mgarciasilva@salud.madrid.org

Dr. Alfons Macaya Ruiz
Affiliation: University Hospital Vall d'Hebron, Barcelona 08035, Spain
Email: amacaya@vhebron.net

Prof. Barbara Garavaglia
Affiliation: IRCCS Foundation, Neurological Institute “Carlo Besta”, Molecular Neurogenetics, 20126 Milan, Italy
Email: segr.neurogenetica@istituto-besta.it

Dr. Eugenia Borgione
Affiliation: Laboratorio di Neuropatologia Clinica, U.O.S. Malattie, Neuromuscolari Associazione OASI Maria SS. ONLUS – IRCCS, Via Conte Ruggero 73, 94018 Troina, Italy
Email: eborgione@oasi.en.it

Dr. Simona Portaro
Affiliation: IRCCS Centro Neurolesi "Bonino Pulejo", SS113, c.da Casazza, 98124 Messina, Italy
Email: simonaportaro@hotmail.it

Dr. Benigno Monteagudo Sanchez
Affiliation: Hospital Arquitecto Marcide, Avenida de la Residencia S/N, Ferrol (A Coruña), 15401 Spain
Email: benims@hotmail.com

Dr. Richard Boles
Affiliation: Courtagen Life Sciences, 12 Gill Street Suite 3700, Woburn, MA 01801 USA
Email: Richard.Boles@courtagen.com

Prof. Savvas Papacostas
Affiliation: Neurology Clinic B, The Cyprus Institute of Neurology and Genetics, 6 International Airport Road, 1683 Nicosia, Cyprus
Email: savvas@cing.ac.cy

Dr. Michail Vikelis
Affiliation: Iatreio Kefalalgias Glyfadas, 8 Lazaraki str, 3rd floor, 16675, Athens, Greece
Email: mvikelis@headaches.gr

Prof James Rothman
Affiliation: Department of Cell Biology, Yale School of Medicine, New Haven, CT
Email: jrothman77@yahoo.com

Prof Dimitri Kullmann
Affiliation: University College London, London, UK

Email: d.kullmann@ucl.ac.uk

Prof Eleni Zamba Papanicolaou
Affiliation: The Cyprus Institute of Neurology & Genetics, Nicosia, Cyprus
Email: ezamba@cing.ac.cy

Dr Efthymios Dardiotis
Affiliation: UNIVERSITY HOSPITAL OF LARISSA, NEUROLOGY Department, Greece
Email: edar@med.uth.gr

Prof Shazia Maqbool
Affiliation: Department of Developmental and Behavioral Pediatrics, CH&ICH, Lahore, Pakistan
Email: drshazimaq@yahoo.com

Prof Shahnaz Ibrahim
Affiliation: Department of Pediatrics and child health, Aga Khan University, Karachi, Pakistan
Email: shahnaz.ibrahim@aku.edu

Prof Salman Kirmani
Affiliation: Department of Paediatrics & Child Health, The Aga Khan University, Karachi , Pakistan
Email: salman.kirmani@aku.edu

Dr. Nuzhat Noureen Rana
Affiliation: Department of Paediatric Neurology, Children Hospital Complex and ICH, Multan, Pakistan
Email: drnuzhatrana@gmail.com

Dr. Osama Atawneh
Affiliation: Hilal Pediatric Hospital Hebron, Hebron West Bank, Palestine
Email: osamaat@gmail.com

Prof Shen-Yang Lim
Affiliation: Department of Biomedical Science, Faculty of Medicine, University of Malaya, Malaysia
Email: limshenyang@gmail.com

Dr Farooq Shaikh
Affiliation: Jeffrey Cheah School of Medicine and Health Sciences, Monash University Malaysia
Email: farooq.shaikh@monash.edu

Prof George Koutsis
Dr Marianthi Breza
Affiliation: Neurogenetics Unit, Neurology Department, Eginition Hospital, National and Kapodistrian University, Athens, Greece
Email: marianthibr@med.uoa.gr

Prof Salvatore Mangano
Affiliation: Unità di Neuropsichiatria Infantile, AOUP "P.Giaccone" Palermo, Italy
Email: salvatore.mangano@unipa.it

Dr Carmela Scuderi
Affiliation: Associazione Oasi Maria SS, 94018 Troina, Italy
Email: cscuderi@oasi.en.it

Dr Eugenia Borgione
Affiliation: Associazione Oasi Maria SS, 94018 Troina, Italy
Email: eborgione@oasi.en.it

Dr Giovanna Morello
Affiliation: Institute of Neurological Sciences, National Research Council, Mangone, Italy
Email: g.morello@isn.cnr.it

Dr Tanya Stojkovic
Affiliation: Institute of Myology, Hôpital La Pitié Salpêtrière, Paris, France
Email: stojkovic.tanya@aphp.fr

Prof Massimi Zollo
Affiliation: CEINGE, Biotecnologie Avanzate S.c.a.rl., Naples, Italy
Email: massimo.zollo@unina.it

Dr Gali Heimer
Affiliation: University Hospital of Tel Aviv, Tel Aviv, Israel
Email: galih.md@gmail.com

Prof Yves A. Dauvilliers
Affiliation: University Hospital Montpellier, Montpellier, France
Email: ydauvilliers@yahoo.fr

Prof Pasquale Striano
Affiliation: Institute "Giannina Gaslini", Genova, Italy
Email: strianop@gmail.com

Dr Issam Al-Khawaja
Affiliation: Albashir University Hospital, Amman, Jordan
Email: isamkhawaja61@gmail.com

Dr Fuad Al-Mutairi
Affiliation: King Saud University, Riyadh, Saudi Arabia
Email: almutairifu@NGHA.MED.SA

Prof Hamed Sherifa
Affiliation: Assiut University Hospital, Assiut, Egypt
Email: hamed_sherifa@yahoo.com

Dr Menelaos Pipis
Affiliation: MRC Centre for Neuromuscular Diseases, UCL Queen Square Institute of Neurology, London, UK.
Email: m.pipis@ucl.ac.uk

Dr Conceicao Bettencourt
Affiliation: Department of Clinical and Movement Neurosciences, UCL Queen Square Institute of Neurology , Queen Square Brain Bank for Neurological Disorders, London WC1N 1PJ
Email: c.bettencourt@ucl.ac.uk

Dr Simon Rinaldi
Affiliation: Nuffield Department of Clinical Neurosciences, University of Oxford & Oxford University

Hospitals NHS Foundation Trust
Email: simon.rinaldi@nhs.net
