# Supplementary material for: Biallelic mutations in neurofascin cause neurodevelopmental impairment and peripheral demyelination
Source: Brain. 2019 Sep 9;142(10):2948–64. doi: 10.1093/brain/awz248 (PMC6763744; doi:10.1093/brain/awz248)

Full unedited gel for Figure 3H : tubulin

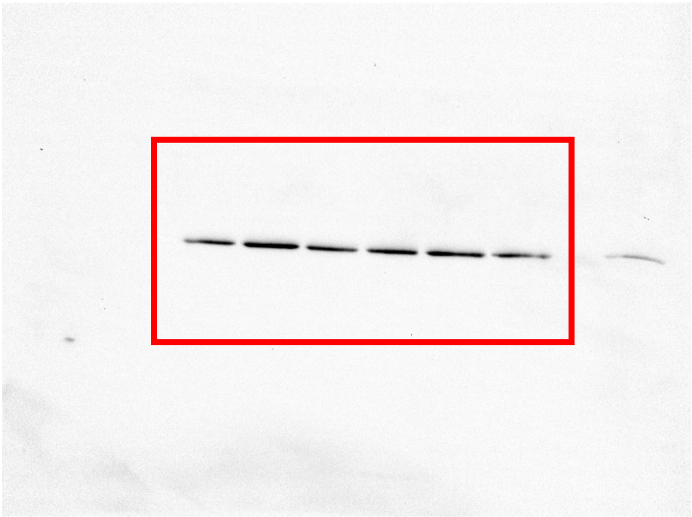

Full unedited gel for Figure 3H : Myc

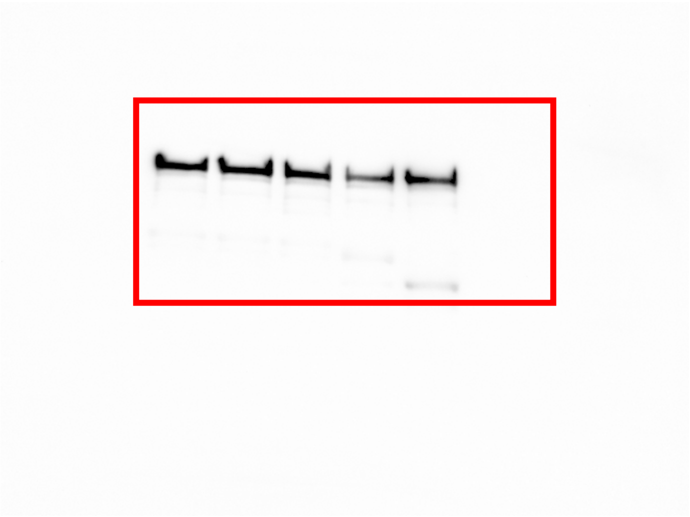

Full unedited gels for Supplemental figure 2 :

Tubulin Myc

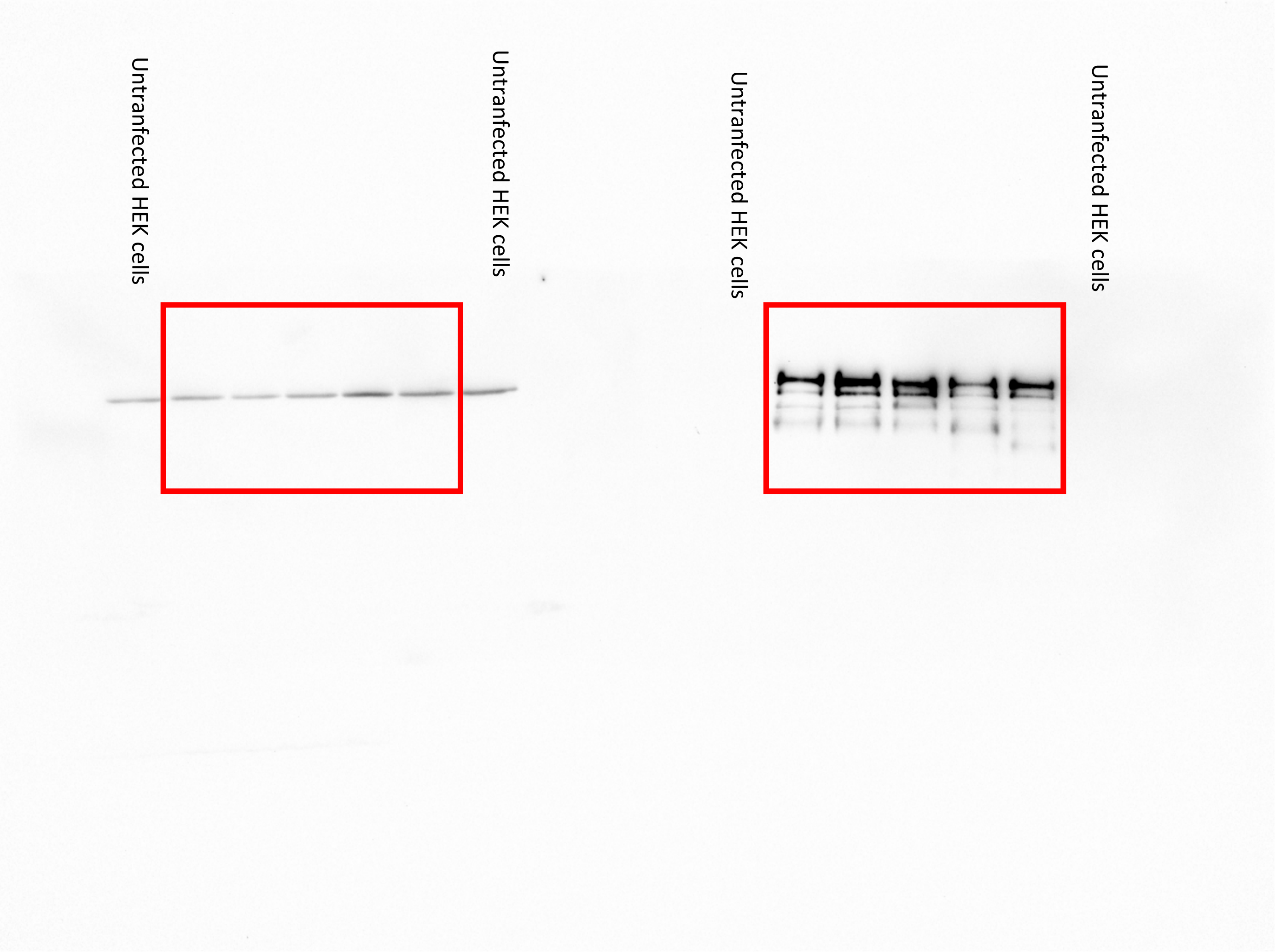

Supplement: awz248_Supplementary_Data [file awz248_supplementary_data.zip › awz248-Suppl_data/Supplementary_Data3.pdf]
