# Supplementary material for: Biallelic mutations in neurofascin cause neurodevelopmental impairment and peripheral demyelination
Source: Brain. 2019 Sep 9;142(10):2948–64. doi: 10.1093/brain/awz248 (PMC6763744; doi:10.1093/brain/awz248)
Supplement: awz248_Supplementary_Data [file awz248_supplementary_data.zip › awz248-Suppl_data/Supplementary_Data4.pdf]

Supplementary Material

Supplementary Video 1. First part of the video shows the hypotonia of patient 1 carrying the p.P694T mutation in *NFASC*, and the second part consists of the neurological examination, showing a complex phenotype of demyelinating neuropathy with global developmental delay, severe hypotonia and some distinctive dysmorphic features. There is no history of seizures but the boy has never acquired any milestones such as walking. Speech is absent and some autistic features are present.

Patient Phenotypes

Proband 1 is a 4 year old boy of North African origin, with first degree consanguineous parents, presented with neonatal hypotonia and psychomotor delay, and some distinctive dysmorphic features. On examination (Video 1), he appeared to be floppy, with very limited muscle strength leaving him unable to walk and with severe dysarthria. Nerve conduction studies (NCS) showed a reduction of the conduction velocity with a near normal amplitude (median: 4.7mV; ulnar: 6.3mV; tibial: 9.4mV). This is likely related to a primarily myelin pathology rather than the loss of large axons fibres. Babinski sign was bilaterally positive, indicating a central nervous system involvement of the upper motor neuron. He is unable to walk. Diagnostic study of the MECP2 gene known to cause Rett Syndrome with multiplex ligation-dependent probe amplification (MLPA) showed no deletion or duplication of the gene ruling out a molecular anomaly of the MECP2 gene as confounding reason for his phenotype.

Proband 2 is an 11 month old boy and was born at 34 weeks gestation. He was initially evaluated shortly after birth regarding complications of global hypotonia, chronic respiratory failure status post tracheostomy and ventilator dependence, feeding dysfunction status post gastrostomy tube placement, and autonomic dysregulation. MRI studies showed mild white matter brain volume loss with prominence size of the lateral ventricles. A testicular ultrasound showed both testicles lying in the inguinal canal with otherwise normal appearance. He underwent an ABR and ear tube placement in his first year. ABR indicated profound bilateral sensorineural hearing loss. A recent echocardiogram suggested a small secundum ASD but otherwise normal. He has not undergone an EMG to date.

Proband 3 is a10 year old female and has been followed for several years at this point. She was born at 34 weeks gestation and was noted to have hypotonia at birth. Her history includes marked cerebral atrophy and a congenital hypomyelinating polyneuropathy. She has startle-induced myoclonus and possibly tonic seizures in the past. Developmentally, she moves her fingers and will move her tongue. She is wheelchair dependent and has spasticity of all four extremities. She is tracheostomy and gastrostomy tube dependent. Recent MRI studies showed interval progression of the dilated ventricles and subarachnoid CSF spaces, with further loss of cerebral white matter, resulting in severe atrophy of the corpus callosum and brainstem. An EMG showed evidence of a severe demyelinating neuropathy with a minor axonal component, consistent with a congenital hypomyelinating syndrome. The EEG

study was abnormal and showed moderate diffuse generalized background slowing indicative of moderate diffuse encephalopathy. The echocardiogram showed a small PFO/ASD and mild physiologic peripheral branch pulmonary stenosis.

Proband 4 is a 4.5 months old boy from a consanguineous Iraqi family presented with severe neurodevelopmental phenotype including hypotonia at birth, global developmental delay, microcephaly, epilepsy and a clinical diagnosis of cerebral palsy (CP). Weight at birth was 3.4 kg and head circumstance at birth was 34.5cm. EEG record at the age of 3 days was within normal limits but could not fully exclude epilepsy. However, EEG performed at the age of 2 months was abnormal showing epileptic activity. His extended medical history revealed bronchopneumonia confirmed by chest X-ray examination at the age of 3 months. An echocardiography performed at birth detected a small PFO but was otherwise normal. CT scan at the age of 1 day did not identify any intracerebral haemorrhage or focal lesions with normal ventricle width. Ultrasound of abdomen revealed normal size and texture of liver, normal biliary system, normal size of spleen and also normal kidney and bladder. He also had a 4 year old sister with global developmental delay (GDD), microcephaly, epilepsy, growth deficiency and cerebral palsy, and being unable to walk.

Proband 5 is a 12 year old boy presenting with hypotonia at 6 months of age and subsequent cerebellar symptoms and bradykinesia but without dystonia. His examination was also noteworthy for areflexia with weakness of lower limbs and subsequent diagnosis of mild intellectual disability. He is ambulant indoors with a walker, attends special education and suffers from mild dysarthria. EMG and NCS at age 6 years were normal except for absent sympathetic skin response in hand and foot upon stimulation of contralateral side. Muscle biopsy was normal.

Proband 6 is a 16 year old boy presented at two month of age with hypotonia. He is the oldest of three siblings, two boys and a girl, born to consanguineous healthy Palestinian parents. Pregnancy, delivery (Term, AGA) and the perinatal course were normal. Initial exam revealed mild truncal and appendicular hypotonia and areflexia. Subsequent motor development was slow with independent ambulation with a walker achieved at 5 years of age. From around 3 years of age cerebellar and extrapyramidal symptoms became apparent with ataxia, dysarthria, dysmetria, bradykinesis and dystonia. Weakness in proximal lower limbs was also noted subsequently. Contractures in lower limbs necessitated bilateral adductor and

hamstring tenotomies at 9 years of age. Despite this progression of symptoms no regression of function has been noted hitherto. Upon entry in school mild cognitive impairment was diagnosed and special education was initiated along with continuation of occupational and physical therapy. Extensive metabolic testing and brain MRI have yielded normal results.

Proband 7 is a 3 year old boy who was born at 34 weeks gestation via an emergency C-Section due to fetal distress. His Apgar scores were 4 and 7 at 1 and 5 minutes, respectively. He has had a respiratory difficulty at birth, and he was subsequently intubated and admitted to NICU. His growth parameters at birth were weight 2.1 kg and head circumference 33.5 cm, and he was noted to have a very small anterior fontanel at birth. He stayed in NICU for 9 months. A tracheostomy was placed for him because he failed extubation several times. He had frequent hospital admissions due to pneumonia and sepsis. There is a positive family history of a similar condition in two cousins and a male sibling who died due to the same condition. His physical examination was significant for hypertelorism, high and wide nasal bridge, micrognathia, glossoptosis, cleft palate, long thin and hyperextensible fingers and hypotonia. Other examinations were within normal limits. Brain MRI showed diffuse white matter T2 hyperintensity. Skeletal survey showed 11 pairs of ribs. EMG, NCS, echocardiography, karyotyping, CGH microarray, CK assay, neonatal screen, and urine organic acids were within normal limits.

Proband 8 was a 4 month girl (at the time of death) who presented during neonatal period with congenital stridor and generalized hypertonia. Her birth weight was 3.2 kg, and she showed poor weight gain since birth even though she was on NG-tube feeding. Developmentally, she has a global developmental delay affecting all domains. She shows no purposeful movements and has a prolonged grasp reflex. She does not have a social smile, but she can babble and coo. There is a family history of a similar condition in two siblings; a brother who died at the age of 3 months and an older sister who is currently 21 months with GDD and hypertonia. There is also a paternal cousin with similar presentation. On examination, she has a non-expressive face with subtle dysmorphism and mild positional deformity of the chest wall. Her current growth parameters at 3 months of age were weight 3.2 kg, length 53 cm and head circumference 36.5 cm all being below the third percentile. On neurological examination, there is a generalized hypertonia affecting upper limbs more than lower limbs and hyperreflexia without a clonus. Hearing and vision were intact, and other examinations were within normal limits.

Proband 9 is a 21-year-old female of Algerian origin, presented with neonatal hypotonia and developmental delay. She presented with a cerebellar syndrome with dysmetria, dysarthria, ataxia and orofacial dyspraxia. She was never able to walk autonomously and after using a walker she is now wheelchair dependent. At clinical examination, she also presented with pyramidal syndrome with brisk reflexes, abnormal oculomotricity, nystagmus, strabismus, hypersalivation, and neuropathic symptoms with cold extremities, hollow feet and wrist drop. Brain MRI at 8 years old identified a cerebellar atrophy as well as hypersignals in the posterior limb of the internal capsule without brainstem anomaly. EMG study performed at 15 years old indicated a motor and sensory neuropathy. She had speech delay and pronounced her first words at the age of 4-year-old. To date, she says only a few words and is in a medical-educational institute. She has an anti-epileptic treatment (Keppra) since age 17 years for a few generalized seizures. EEG was abnormal with the presence of rhythmic polyspike-wave discharges without clinical manifestation. Extensive metabolic and diagnostic work-up was normal.

Proband 10 is a 16-year-old male of Algerian origin, and the brother of proband 9. He has a less severe phenotype compared to his sister. His first symptoms appeared at the age of 1.5 year. At clinical examination, he presented a cerebellar syndrome associated with ataxia and dysarthria, jerky eye pursuit, nystagmus, and signs suggesting a neuropathy with cold, hollow feet and wrist drop. He can walk with a walker and autonomous in daily activities. His extensive metabolic and diagnostic work-up showed normal results. EMG study performed at 10 years old indicated a motor and sensitive axonal neuropathy. Brain MRI at 2 years old was normal but a subsequent MRI at the age of 10 years also revealed a cerebellar atrophy with hyperintensities in the posterior limb of the internal capsule, but without brainstem anomaly.

Genetic analyses, variant calling, filtering and interpretation

In the index family (Family 1) homozygosity analysis was performed from trio-WES data using web-based tools (www.homozygositymapper.org) and identified different regions of homozygosity in the proband in chromosome 1 (186919863-200312546; 200550243-205272722 ; 245530282-248604452), chromosome 2 (52929780-65200618; 69688727-72742083; 73280303-89890648), chromosome 4 (4239539-7312145), chromosome 9 (139440852-139973629), chromosome 16 (1306986-3077710; 3119304-7703785) and chromosome 17 (8007650-10555061) Rare variants with a frequency <0.01% in public

databases (including 1,000 Genomes project and ExAC) present as homozygous in the Proband and heterozygous in the unaffected parents were selected. This restricted our list candidate variants to homozygous variants in *LAD1* (MIM: 602314; NM_005558.3: c.853A>G; p.Thr285Ala), *ZBED6* (MIM: 613512; encoding a transcriptional repressor containing a zinc-finger domain, *USP43* (MIM: 615695; NM_014709.3: c.4706C>T; p.Ala1569Val) and *NFASC* (NM_001005388: c.2080C>A; p.P694T). The homozygous variant identified in *NFASC* affects an highly conserved residue within fibronectin (type III) domain and predicted to be deleterious (Supplementary Fig. 8) and was prioritized based on the biological importance of this gene in the nodal and paranodal organization and the knowledge of demyelinating neuropathy phenotypes (similar to our patient) in patients with IgG4 antibodies against Nfasc155 and Nfasc186 isoforms. Sanger sequencing segregation analysis confirmed segregation and identified the variant as heterozygous in the unaffected sister.

WGS was carried out for Patients 2 at GeneDx (Gaithersburg, USA). Using genomic DNA from the proband and parents of Family 2, PCR-free whole genome sequencing libraries were prepared using Kapa Hyper Prep following the manufacturer's protocol (Kapa Biosystems). Massively parallel (NextGen) sequencing was performed on an Illumina HiSeq4000 with 2x150bp paired-end reads. Reads were aligned to human genome build GRCh37/UCSC hg19, and analysed for sequence variants using a custom-developed analysis tool. Intronic regions, outside of the flanking splice junctions, and regulatory regions, are only analysed for known disease-associated variants. Additional sequencing technology and variant interpretation protocol has been previously described (Retterer *et al.*, 2016). This revealed a homozygous frameshift variant in *NFASC* (NM_015090.3, c.2771delC). The general assertion criteria for variant classification are publicly available on the GeneDx ClinVar submission page (http://www.ncbi.nlm.nih.gov/clinvar/submitters/26957/). Variants were confirmed in all family members by Sanger sequencing.

Genomic DNA was taken from Family 3 according to the ethics approval of Mashhad University (IR.MUMS.REC.1395.534) and samples were processed in Nijmegen under the Diagnostic Innovation programme. The index case (patient 3) was subjected to WES at Novogene, Hongkong on an Illumina HiSeq 2500. Exome capture was performed using Agilent SureSelect Human All Exon V6 Kit, sequencing was undertaken on the Illumina HiSeq 2500 Genome Analyzer machine. Sequencing depth was 50× using paired-end

sequencing resulting in sequences of 150 bases from each end of the fragments. UCSC hg19 was used as a reference genome. VarScan version 2.2.5 and MuTec and GATK Somatic Indel Detector were used to detect SNV and InDels, respectively. Data was filtered for MAF < 1% in public control databases such as dbSNP, ExAc and gnomAD (gnomAD, http://gnomad.broadinstitute.org). Subsequently, variants occurring with MAF > 1% in the Iranome (http://www.iranome.ir) were also excluded. The remaining variants were filtered for known disease causing genes with emphasis on diseases compatible with the patient phenotype (neurodevelopmental disease) and homozygous variants were prioritised due to the autosomal recessive inheritance pattern of disease and known consanguinity in the family. This revealed a homozygous missense variant in *NFASC* (NM_001005388, c.388A>G; p.N130D).

DNA from Family 4 was extracted from peripheral blood cells. Homozygosity mapping was done with DNA samples of the index case, two affected siblings and one unaffected sibling using the GeneChip Human Mapping 6.0 SNP array (Affymetrix). Using HomozygosityMapper, we delineated three genetic intervals (chr1:200431643-214287420 ; chr3:4807550-13273299 ; chr8:135871032-136836216 (GRCh37) comprising 23.3 Mbp) that were homozygous only in all three affected individuals, but not in the unaffected sibling (Seelow and Schuelke, 2012). Only variants within the homozygous regions were considered pathogenic. Exonic sequences were enriched from the index patient using the SureSelect V4 Human All Exon 51 Mb Kit (Agilent), and sequenced on a HiSeq2000 machine (Illumina). Whole exome sequencing (WES) achieved a mean coverage of 90.6x with 92.9% of the RefSeq sequences covered >3x and 86.8% >10x. FASTQ-files were aligned to the human GRCh37.p11 (hg19) reference sequence using the BWA-MEM V.0.7.1 aligner. A variant file was generated for all exons±20 bp flanking regions using the GATK V.3.8 software package (McKenna *et al.*, 2010; DePristo *et al.*, 2011), and sent to VariantTaster2 (http://www.varianttaster.org) for assessment of potential pathogenicity (Schwarz *et al.*, 2014). Filtering options were used as described (Renesse *et al.*, 2014). All relevant variants were inspected visually using the Integrative Genomics Viewer (http://www.broadinstitute.org/igv/). Within the homozygous intervals only the homozygous chr1:204,951,136T>C *NFASC* variant was predicted to be pathogenic by the VariantTaster2 software. It was absent in the ExAC and gnomAD databases.

Genotype–phenotype segregation of the *NFASC* variant in the family was verified by automatic Sanger sequencing using the BigDye (Applied Biosystems) protocol on an

ABI3500 Genetic Analyzer (Applied Biosystems). Exon 20 of *NFASC* (NM_015090) was amplified with the primer pair FW: 5-cagcctgtctgtccctctg-3, REV 5-acggggaggagactactcac-3.

Family 5 was subjected to WES as described previously (Anazi *et al.*, 2017); family 6 was subjected to WES as described previously (Chemin *et al.*, 2018) and was identified during the replication cohort screening of exomes from 40 undiagnosed individuals with undiagnosed autosomal recessive neuropathies.

Molecular Modelling

The WT *NFASC*, as well as the two variants N130D and R359P were built from structure PDB ID 3P3Y. While the transcript in which the N130D mutant is similar to the sequence of the crystallographic structure used in this study, the transcript in which the R359P mutant is different. However, for lacking of a more similar crystallographic structure and considering that the differences are located at a considerable distance from the mutation site, the same starting structure was used to model the structure of the R359P mutant. Each protein was then placed in a cubic box and minimised.

Consortia and networks involved in this study

The Synaptopathies and Paroxysmal Syndromes (SYNaPS) Study Group

(http://neurogenetics.co.uk/synaptopathies-synaps/)

Study Group Members:

Prof Stanislav Groppa
Affiliation: Department of Neurology and Neurosurgery, Institute of Emergency Medicine, Chisinau, Republic of Moldova.
Email: sgroppa@gmail.com

Dr. Blagovesta Marinova Karashova
Affiliation: Department of Paediatrics, Medical University of Sofia, Sofia 1431, Bulgaria
Email: blagovestakarashova@gmail.com

Prof Lionel Van Maldergem
Affiliation: Centre of Human Genetics, University Hospital Liege, Liege 4000, Belgium
Email: lvanmaldergem@chu-besancon.fr

Dr. Wolfgang Nachbauer
Affiliation: Department of Neurology, Medical University Innsbruck, Anichstrasse 35, Innsbruck 6020, Austria
Email: Wolfgang.Nachbauer@i-med.ac.at

Prof. Sylvia Boesch
Affiliation: Department of Neurology, Medical University Innsbruck, Anichstrasse 35, Innsbruck 6020, Austria
Email: sylvia.boesch@i-med.ac.at

Dr. Larissa Arning
Affiliation: Department of Human Genetics, Ruhr-University Bochum, Bochum 44801, Germany
Email: Larissa.Arning@ruhr-uni-bochum.de

Prof. Dagmar Timmann
Affiliation: Braun Neurologische Universitätsklinik Universität Essen, Hufelandstr 55, Essen D-45122, Germany
Email: Dagmar.Timmann-Braun@uni-duisburg-essen.de

Prof. Bru Cormand
Affiliation: Department of Genetics, Universitat de Barcelona, Barcelona 08007, Spain
Email: bcormand@ub.edu

Dr. Belen Pérez-Dueñas
Affiliation: Hospital Sant Joan de Deu, Esplugues de Llobregat 08950, Barcelona, Spain
Email: bperez@sjdhospitalbarcelona.org

Dr Gabriella Di Rosa, MD, PhD
Affiliation: Department of Pediatrics, University of Messina, Messina 98123, Italy
Email: gdirosa@unime.it

Prof. Jatinder S. Goraya, MD, FRCP
Affiliation: Division of Paediatric Neurology, Dayanand Medical College & Hospital, Ludhiana, Punjab 141001, India
Email: gorayajs@gmail.com

Prof. Tipu Sultan
Affiliation: Division of Paediatric Neurology, Children's Hospital of Lahore, Lahore 381-D/2, Pakistan
Email: tipusultanmalik@hotmail.com

Prof Jun Mine
Affiliation: Department of Paediatrics, Shimane University, Faculty of Medicine, Izumo, 693-8501, Japan
Email: jmine@med.shimane-u.ac.jp

Prof. Daniela Avdjieva,
Affiliation: Department of Paediatrics, Medical University of Sofia, Sofia 1431, Bulgaria
Email: davdjieva@yahoo.com

Dr. Hadil Kathom,
Affiliation: Department of Pediatrics, Medical University of Sofia, Sofia 1431, Bulgaria
Email: hadilmk@gmail.com

Prof.Dr Radka Tincheva
Affiliation: Head of Department of Clinical Genetics, University Pediatric Hospital, Sofia 1431, Bulgaria
Email: radka.tincheva@gmail.com

Prof. Selina Banu
Affiliation: Neurosciences Unit, Institute of Child Health and Shishu Shastho Foundation Hospital, Mirpur, Dhaka 1216, Bangladesh
Email: selinabanu17@gmail.com

Prof. Mercedes Pineda-Marfa
Affiliation Servei de Neurologia Pediàtrica, l'Hospital Universitari Vall d'Hebron, Barcelona 08035, Spain
Email: pineda@hsjdbcn.org

Prof. Pierangelo Veggiotti
Affiliation: Unit of Infantile Neuropsychiatry Fondazione
Istituto Neurologico "C. Mondino" IRCCS, Via Mondino 2, Pavia 27100, Italy
Email: pierangelo.veggiotti@unipv.it

Prof. Michel D. Ferrari
Affiliation: Leiden University Medical Center, Albinusdreef 2, Leiden 2333, Netherlands
Email: M.D.Ferrari@lumc.nl

Prof Arn M J M van den Maagdenberg
Affiliation: Leiden University Medical Center, Albinusdreef 2, Leiden 2333, Netherlands
A.M.J.M.van_den_Maagdenberg@lumc.nl

Prof. Alberto Verrotti
Affiliation: University of L'Aquila, L'Aquila, Italy
Email: verrottidipianella@univaq.it

Prof Giangluigi Marseglia
Affiliation: Department of Pediatrics, University of Pavia, IRCCS Policlinico "San Matteo", Pavia 27100, Italy
Email: gl.marseglia@smatteo.pv.it

Dr. Salvatore Savasta
Affiliation: Division of Pediatric Neurology, Department of Pediatrics, University of Pavia, IRCCS Policlinico "San Matteo", Pavia 27100, Italy
Email: S.Savasta@smatteo.pv.it

Dr. Mayte García-Silva
Affiliation: Hospital Universitario 12 de Octubre, Madrid 28041, Spain
Email: mgarciasilva@salud.madrid.org

Dr. Alfons Macaya Ruiz
Affiliation: University Hospital Vall d'Hebron, Barcelona 08035, Spain
Email: amacaya@vhebron.net

Prof. Barbara Garavaglia
Affiliation: IRCCS Foundation, Neurological Institute "Carlo Besta", Molecular Neurogenetics, 20126 Milan, Italy
Email: segr.neurogenetica@istituto-besta.it

Dr. Eugenia Borgione
Affiliation: Laboratorio di Neuropatologia Clinica, U.O.S. Malattie, Neuromuscolari Associazione OASI Maria SS. ONLUS – IRCCS, Via Conte Ruggero 73, 94018 Troina, Italy
Email: eborgione@oasi.en.it

Dr. Simona Portaro
Affiliation: IRCCS Centro Neurolesi "Bonino Pulejo", SS113, c.da Casazza, 98124 Messina, Italy
Email: simonaportaro@hotmail.it

Dr. Benigno Monteagudo Sanchez
Affiliation: Hospital Arquitecto Marcide, Avenida de la Residencia S/N, Ferrol (A Coruña), 15401 Spain
Email: benims@hotmail.com

Dr. Richard Boles
Affiliation: Courtagen Life Sciences, 12 Gill Street Suite 3700, Woburn, MA 01801 USA
Email: Richard.Boles@courtagen.com

Prof. Savvas Papacostas
Affiliation: Neurology Clinic B, The Cyprus Institute of Neurology and Genetics, 6 International Airport Road, 1683 Nicosia, Cyprus
Email: savvas@cing.ac.cy

Dr. Michail Vikelis
Affiliation: Iatreio Kefalalgias Glyfadas, 8 Lazaraki str, 3rd floor, 16675, Athens, Greece
Email: mvikelis@headaches.gr

Prof James Rothman
Affiliation: Department of Cell Biology, Yale School of Medicine, New Haven, CT
Email: jrothman77@yahoo.com

Prof Dimitri Kullmann
Affiliation: University College London, London, UK
Email: d.kullmann@ucl.ac.uk

Prof Eleni Zamba Papanicolaou
Affiliation: The Cyprus Institute of Neurology & Genetics, Nicosia, Cyprus
Email: ezamba@cing.ac.cy

Dr Efthymios Dardiotis
Affiliation: UNIVERSITY HOSPITAL OF LARISSA, NEUROLOGY Department, Greece
Email: edar@med.uth.gr

Prof Shazia Maqbool
Affiliation: Department of Developmental and Behavioral Pediatrics, CH&ICH, Lahore, Pakistan
Email: drshazimaq@yahoo.com

Prof Shahnaz Ibrahim
Affiliation: Department of Pediatrics and child health, Aga Khan University, Karachi, Pakistan
Email: shahnaz.ibrahim@aku.edu

Prof Salman Kirmani
Affiliation: Department of Paediatrics & Child Health, The Aga Khan University, Karachi , Pakistan
Email: salman.kirmani@aku.edu

Dr. Nuzhat Noureen Rana
Affiliation: Department of Paediatric Neurology, Children Hospital Complex and ICH, Multan, Pakistan
Email: drnuzhatrana@gmail.com

Dr. Osama Atawneh
Affiliation: Hilal Pediatric Hospital Hebron, Hebron West Bank, Palestine
Email: osamaat@gmail.com

Prof Shen-Yang Lim

Affiliation: Department of Biomedical Science, Faculty of Medicine, University of Malaya, Malaysia
Email: limshenyang@gmail.com

Dr Farooq Shaikh
Affiliation: Jeffrey Cheah School of Medicine and Health Sciences, Monash University Malaysia
Email: farooq.shaikh@monash.edu

Prof George Koutsis
Dr Marianthi Breza
Affiliation: Neurogenetics Unit, Neurology Department, Eginition Hospital, National and Kapodistrian University, Athens, Greece
Email: marianthibr@med.uoa.gr

Prof Salvatore Mangano
Affiliation: Unità di Neuropsichiatria Infantile, AOUP "P.Giaccone" Palermo, Italy
Email: salvatore.mangano@unipa.it

Dr Carmela Scuderi
Affiliation: Associazione Oasi Maria SS, 94018 Troina, Italy
Email: cscuderi@oasi.en.it

Dr Eugenia Borgione
Affiliation: Associazione Oasi Maria SS, 94018 Troina, Italy
Email: eborgione@oasi.en.it

Dr Giovanna Morello
Affiliation: Institute of Neurological Sciences, National Research Council, Mangone, Italy
Email: g.morello@isn.cnr.it

Dr Tanya Stojkovic
Affiliation: Institute of Myology, Hôpital La Pitié Salpêtrière, Paris, France
Email: stojkovic.tanya@aphp.fr

Prof Massimi Zollo
Affiliation: CEINGE, Biotecnologie Avanzate S.c.a.rl., Naples, Italy
Email: massimo.zollo@unina.it

Dr Gali Heimer
Affiliation: University Hospital of Tel Aviv, Tel Aviv, Israel
Email: galih.md@gmail.com

Prof Yves A. Dauvilliers
Affiliation: University Hospital Montpellier, Montpellier, France
Email: ydauvilliers@yahoo.fr

Prof Pasquale Striano
Affiliation: Institute “Giannina Gaslini”, Genova, Italy
Email: strianop@gmail.com

Dr Issam Al-Khawaja
Affiliation: Albashir University Hospital, Amman, Jordan
Email: isamkhawaja61@gmail.com

Dr Fuad Al-Mutairi
Affiliation: King Saud University, Riyadh, Saudi Arabia
Email: almutairifu@NGHA.MED.SA

Prof Hamed Sherifa
Affiliation: Assiut University Hospital, Assiut, Egypt
Email: hamed_sherifa@yahoo.com

Dr Menelaos Pipis
Affiliation: MRC Centre for Neuromuscular Diseases, UCL Queen Square Institute of Neurology, London, UK.
Email: m.pipis@ucl.ac.uk

Dr Conceicao Bettencourt
Affiliation: Department of Clinical and Movement Neurosciences, UCL Queen Square Institute of Neurology , Queen Square Brain Bank for Neurological Disorders, London WC1N 1PJ
Email: c.bettencourt@ucl.ac.uk

Dr Simon Rinaldi
Affiliation: Nuffield Department of Clinical Neurosciences, University of Oxford & Oxford University Hospitals NHS Foundation Trust
Email: simon.rinaldi@nhs.net

Supplementary Figures

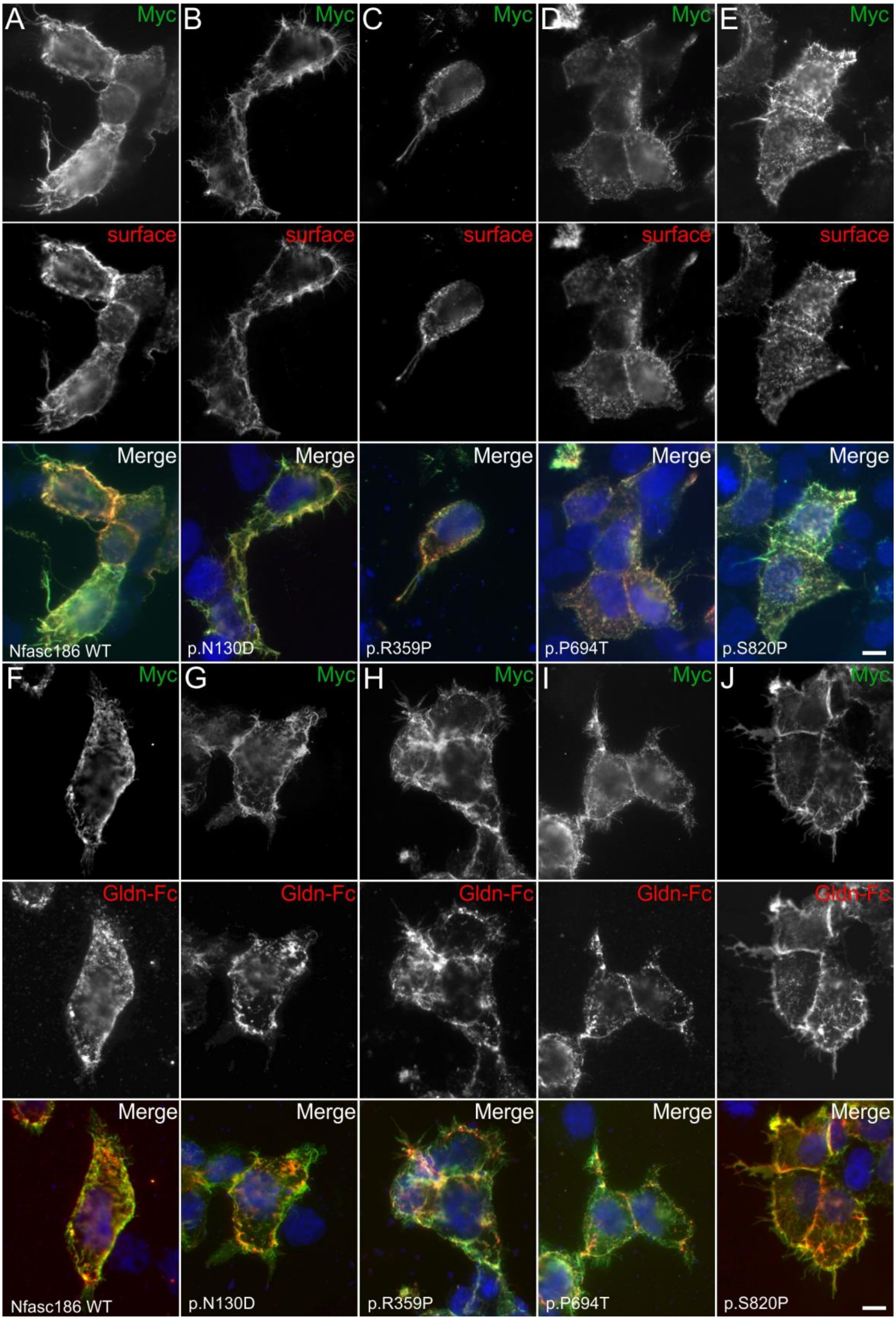

Supplemental Fig. 1. Surface expression of Nfasc186 variants.

A-E. Myc-tagged Nfasc186 variants were expressed in HEK cells and their surface expression was monitored by incubating the live cells with anti-Nfasc186 IgG (red) prior to fixation and permeabilization. Nfasc186 was then revealed using an anti-Myc antibody (green). Nfasc186 variants appeared to be normally targeted to the cell surface. No intracellular retention was observed. **F-J.** Myc-tagged Nfasc186 variants (green) were expressed in HEK cells and the binding to gliomedin (Gldn) was monitored by incubating the live cells with Gldn-Fc proteins (1 µg; red) prior to fixation and permeabilization. Gldn bound similarly to all Nfasc186 variants. Nuclei are stained DAPI (blue). Scale bars: 10 µm.

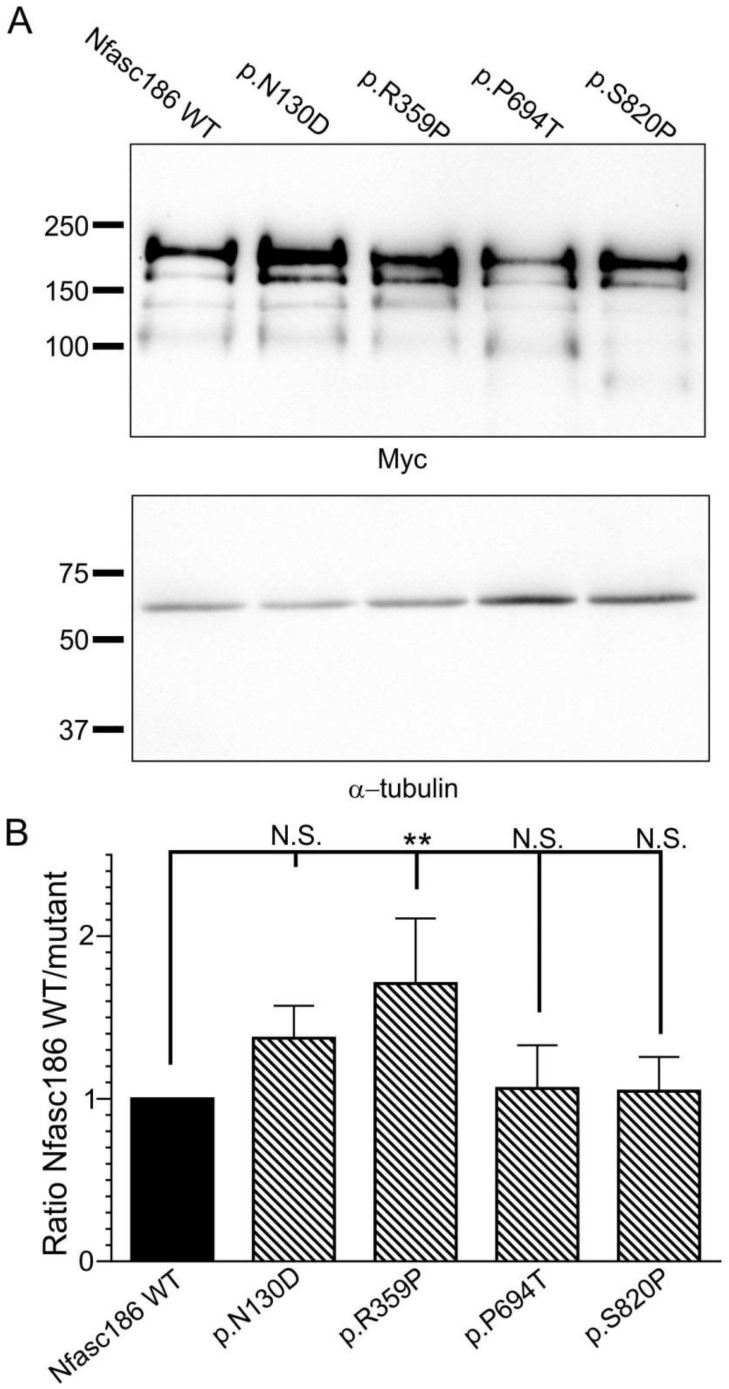

Supplementary Fig. 2. A. Western blot analysis of HEK cells transfected with Nfasc186 variants and revealed with anti-Myc antibodies or anti-a-tubulin antibodies as loading control. Homozygous Nfasc186 variants associated with a severe pathology do not affect Nfasc186 protein level **B.** Protein expression levels were analyzed by normalizing the signals to the corresponding a-tubulin signal, then to WT Nfasc186 in 4 independent experiments. While p.N130D had a higher expression level, the expression levels of p.P694T, p.S820P and p.R359P were normal compared to WT Nfasc186 (Mann-Whitney test). (** $P<0.005$; by unpaired two-tailed Student's t-tests for two samples of equal variance and by one-way

ANOVA followed by Bonferroni's post-hoc tests). Bars represent mean and S.E.M. Molecular weight markers are shown on the left (in kDa). N.S. = not significant.

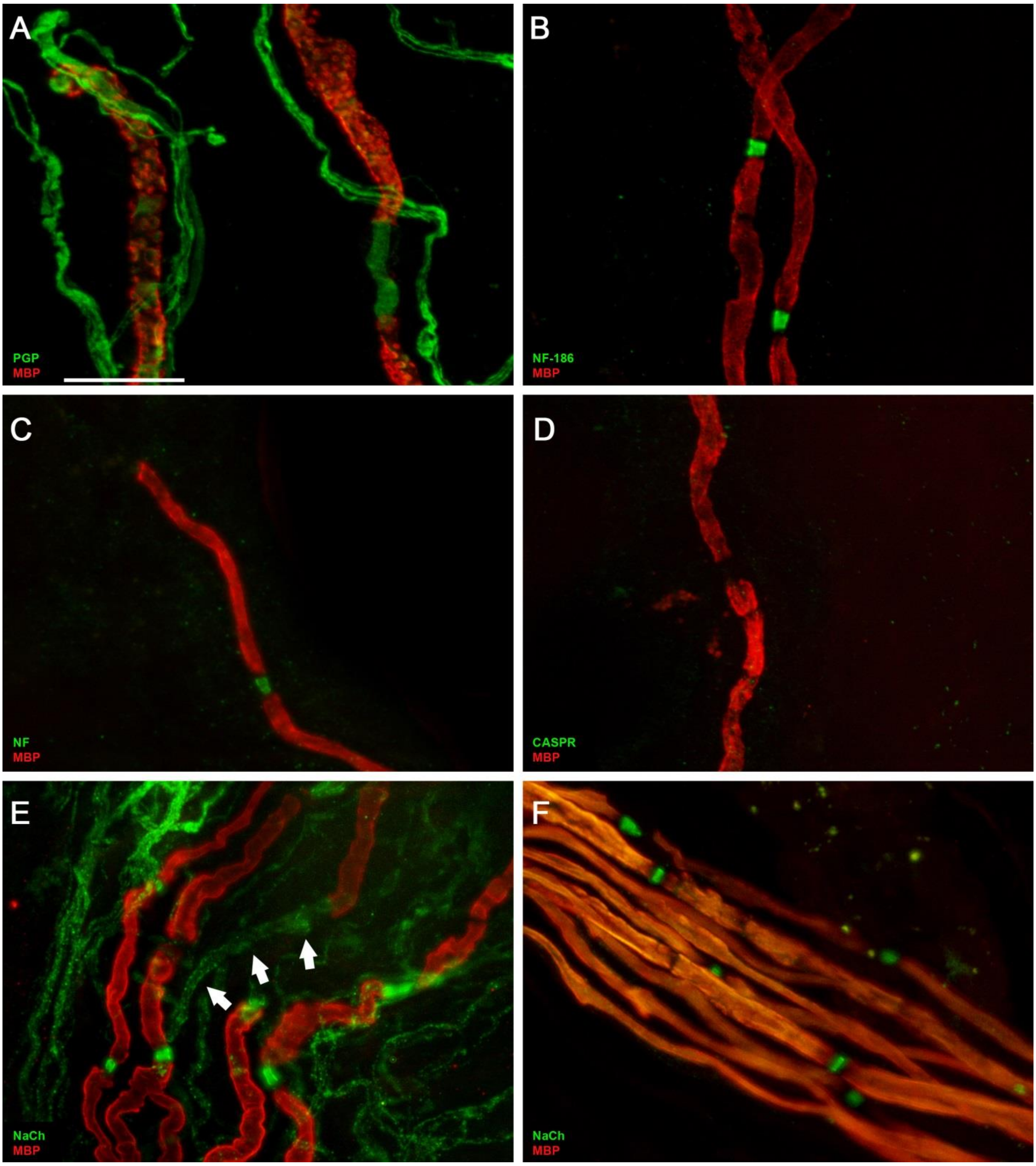

Supplementary Fig. 3. Paranodal alterations in myelinated axons from patient 3. Confocal images of cutaneous innervation from hairy skin of patient 3 showing abnormalities of myelin sheet and enlargement of the nodal gap (3A), normal expression of Nfasc186 staining in the node as evidenced by staining with anti panNeurofascin (NF; 3B) and anti-Nfasc186 antibodies (NF186; 3C), but absent Nfasc155 and CASPR1 immunoreactivity at the paranodal regions (3B and D). The distribution of Nav channels (NaCh) was normal in several nodes that appeared however larger, less compacted than in the control skin (3F) with

a granular distribution of Nav evident along the axonal profiles of large fibres devoid of myelin (3E) that suggests a Nav remodeling after demyelinating phenomena.

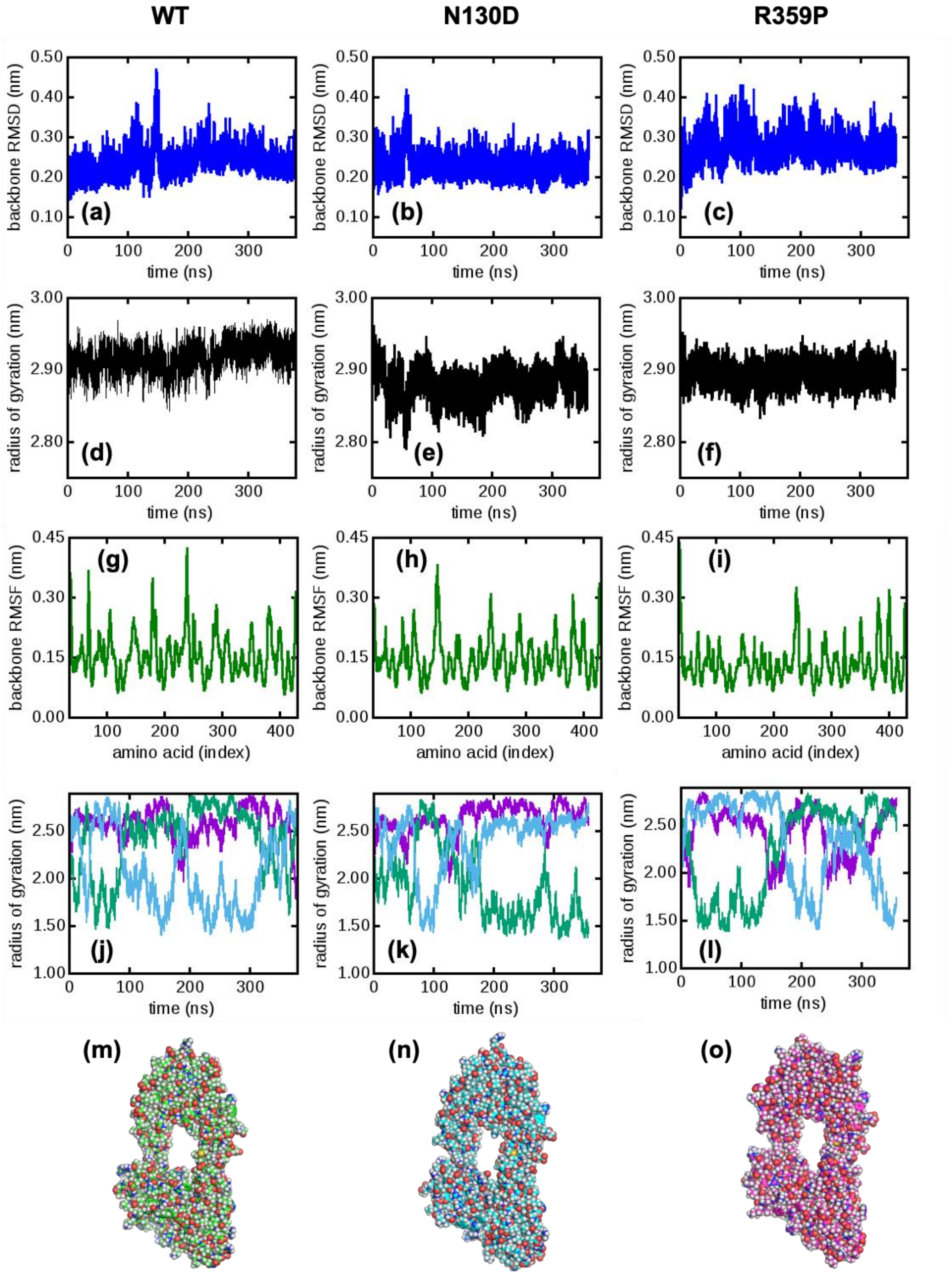

Supplementary Fig. 4. (a-c) Backbone Root Mean Square Deviation (RMSD, blue), (d-f) backbone radius of gyration (black), and (g-i) backbone RMSF (green) for WT Nfasc186 (a,d,g) and its mutants (b,e,h) N130D, and (c,f,i) R359P. Principal axes of the neurofascin

and mass weighted radius of gyration x- y- z- components (purple, green, cyan), for (j) WT Nfasc186 and its mutants (k) N130D and (l) R359P. (m-o) Rearrangement of the Nfasc186 after 200 ns of molecular dynamics simulation for the WT protein and its mutants.

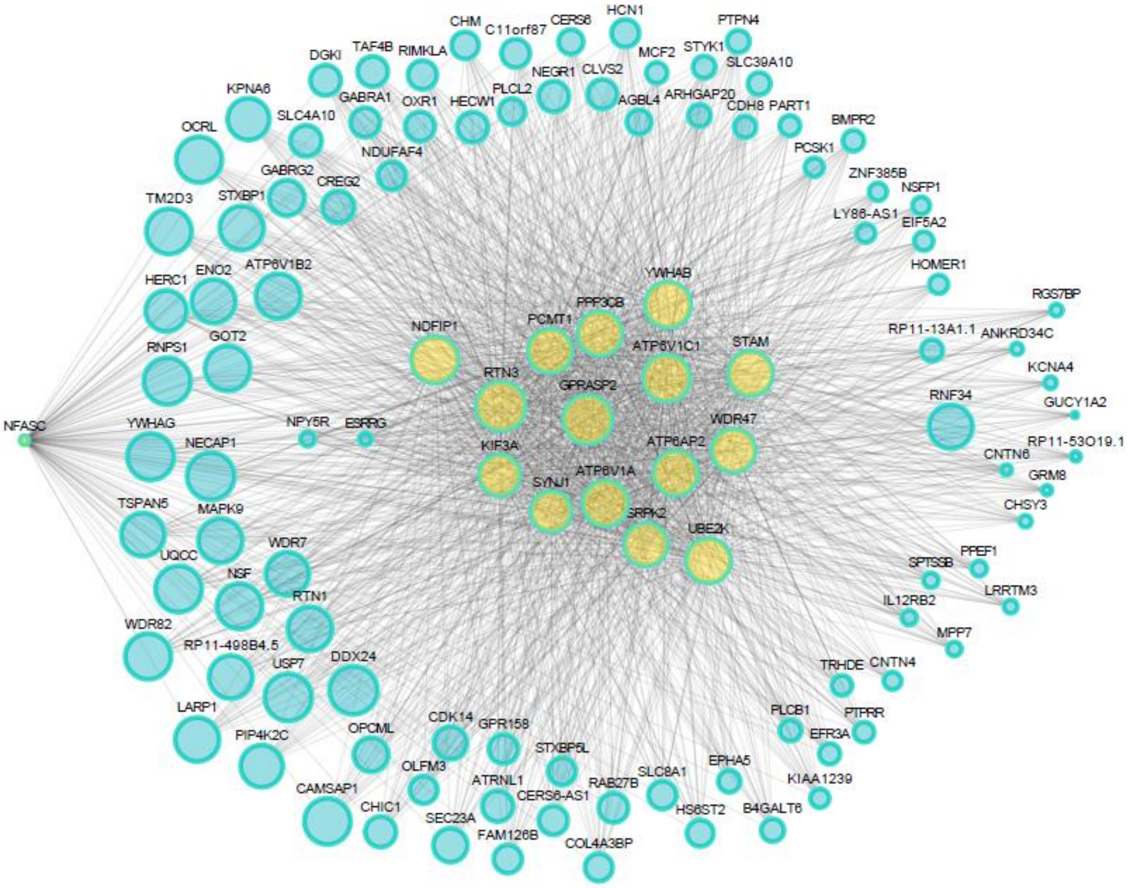

Supplementary Fig. 5. Cell-specific, co-expression network and brain region expression analysis plots. This module (turquoise) in the anterior cingulate cortex is significantly enriched for mental retardation/intellectual disability (ID) genes. 15 of these genes (in yellow) together with *NFASC* (in yellow too) are used as seeds for all other genes within the module connected according to the highest strength to the seeds.

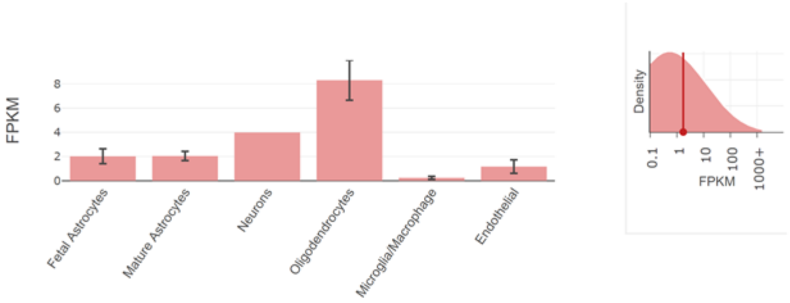

Supplementary Fig. 6. Cell-type specific expression of *NFASC* in human cortex generated using immunopanning (Zhang *et al.*, 2016) (http://www.brainrnaseq.org/)

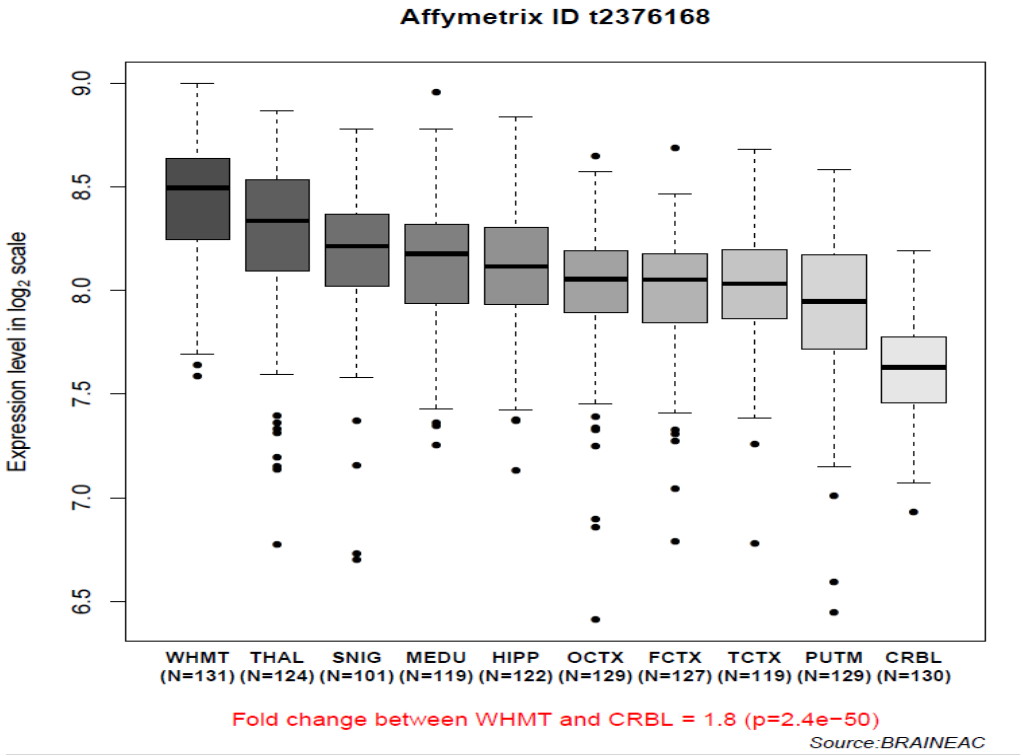

Supplementary Fig. 7. *NFASC* is expressed in various brain regions, with the highest transcript level in white matter (from the BRAINEAC). WHMT, intralobular white matter; THAL, thalamus (at the level of the lateral geniculate nucleus); SNIG, substantia nigra; MEDU, inferior olivary nucleus (sub-dissected from the medulla); HIPP, hippocampus; OCTX, occipital cortex; FCTX, frontal cortex; TCTX, temporal cortex; PUTM, putamen (at the level of the anterior commissure); CRBL, cerebellar cortex.

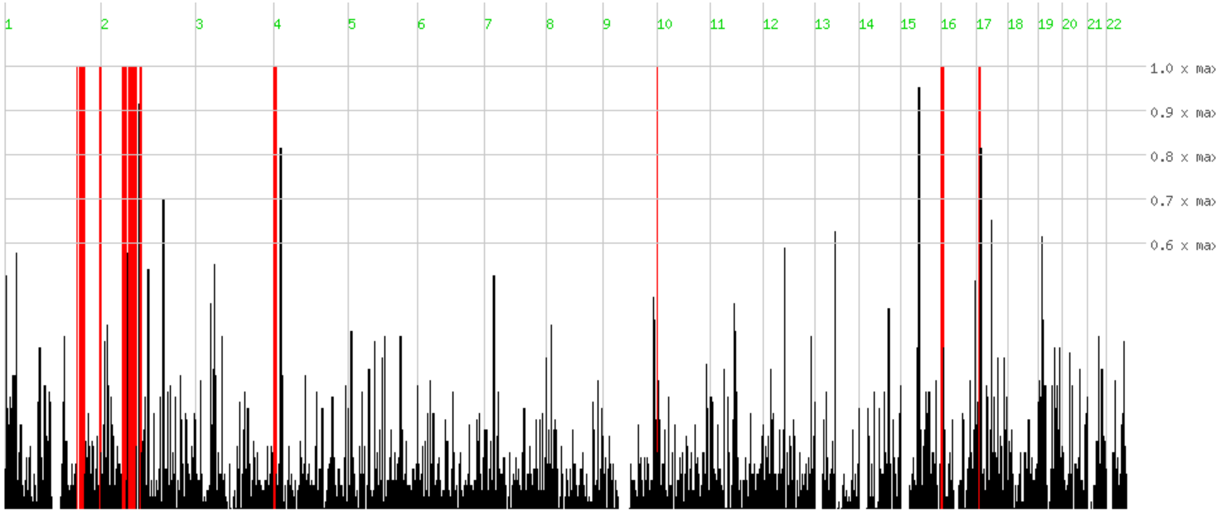

80	1	186919863	200312546	n/a	n/a	region	genotypes
80	1	200550243	205272722	n/a	n/a	region	genotypes
80	1	245530282	248604452	n/a	n/a	region	genotypes
80	2	52929780	65200618	n/a	n/a	region	genotypes
80	2	69688727	72742083	n/a	n/a	region	genotypes
80	2	73280303	89890648	n/a	n/a	region	genotypes
80	2	99779642	103334969	n/a	n/a	region	genotypes
80	4	67857	3589623	n/a	n/a	region	genotypes
80	4	4239539	7312145	n/a	n/a	region	genotypes
80	9	139440852	139973629	n/a	n/a	region	genotypes
80	16	1306986	3077710	n/a	n/a	region	genotypes
80	16	3119304	7703785	n/a	n/a	region	genotypes
80	17	8007650	10555061	n/a	n/a	region	genotypes

Supplementary Fig. 8. Shared regions of homozygosity were identified using Homozygosity Mapper. Homozygosity mapping in patient 1 revealed several homozygous regions and the region (highlighted in yellow) on chromosome 1 includes *NFASC*.
