# Supplementary material for: Biallelic mutations in neurofascin cause neurodevelopmental impairment and peripheral demyelination
Source: Brain. 2019 Sep 9;142(10):2948–64. doi: 10.1093/brain/awz248 (PMC6763744; doi:10.1093/brain/awz248)
Supplement: awz248_Supplementary_Data [file awz248_supplementary_data.zip › awz248-Suppl_data/Supplementary_Data1.pdf]

GTEx Tissue in which network was constructed	Module Membership	Module Name	Module size	FDR-corrected GO term enrichment p-values	Cell type predictions and associated p-values
Tibial nerve	0.8287	purple	296	ensheathment of neurons GO:0007272 (p-value 0.0132), axon ensheathment GO:0008366 (p-value 0.0132)	Oligodendrocyte-External (p-value 1.588e-07).
Amygdala	0.8741	darkgreen	529	oligodendrocyte differentiation GO:0048709 (p-value 2.61e-06), glial cell differentiation GO:0010001 (p-value 6.64e-06), gliogenesis GO:0042063 (p-value 2.22e-05), regulation of gliogenesis GO:0014013 (p-value 0.000256), glial cell development GO:0021782 (p-value 0.00369)	Oligodendrocytes in Human brain Module (Geschwind,2010) (p-value 4.267e-74). Oligodendrocytes in Cortex (p-value 8.137e-33). Oligodendrocytes, definite (Cahoy, 2008) (p-value 2.02e-14). Oligodendrocytes from conservative data set (Lein, 2007) (p-value 4.81e-06). Oligodendrocyte-External (p-value 1.195e-23).
Anterior Cingulate Cortex	0.7396	turquoise	3916	organelle organization GO:0043933 (p-value 5.35e-10), chemical synaptic transmission GO:0099536 (p-value 6.68e-10), mitochondrion organization GO:0061024 (p-value 7.25e-09), protein ubiquitination GO:0006464 (p-value 1.01e-08), protein modification by small protein conjugation or removal GO:0036211 (p-value 1.49e-08)	Neuron in Human brain Module (Geschwind,2010) (p-value 2.599e-108). Neuron module in Cortex (p-value 6.091e-72). Neuron, pyramidal in network from Sugino/Winden (p-value 1.578e-11). Neuron, definite (Cahoy, 2008) (p-value 3.405e-35). Neuron, probably (Cahoy, 2008) (p-value 2.007e-138). Neurons-Cahoy (p-value 0.006484). Neuron_Pyramidal_CA1-External (p-value 1.343e-12). Neuron_Interneuron-External (p-value 2.68e-15). Neuron_Pyramidal_S1-External (p-value 1.828e-07). Neuron_Dopaminergic_SNigra-External (p-value 3.798e-06). Neuron.In1-External (p-value 0.002993). Neuron.Ex2-External (p-value 0.01224). Neuron.Ex4-External (p-value 0.00388). Neuron.Ex7-External (p-value 0.008385).
Caudate	0.7992	black	945	nervous system development GO:0007399 (p-value 6.02e-13), neurogenesis GO:0022008 (p-value 8.88e-12), ensheathment of neurons GO:0007272 (p-value 1.87e-11), axon ensheathment GO:0008366 (p-value 1.87e-11), myelination GO:0042552 (p-value 8.78e-11)	Oligodendrocytes in Human brain Module (Geschwind,2010) (p-value 1.442e-183). Oligodendrocytes in Cortex (p-value 2.687e-97). Oligodendrocytes, definite (Cahoy, 2008) (p-value 1.284e-39). Oligodendrocytes from conservative data set (Lein, 2007) (p-value 3.706e-25). Oligodendrocytes-Cahoy (p-value 1.285e-05). Oligodendrocyte-External (p-value 8.934e-59).
Cerebellar Hemisphere	0.8597	lightcyan1	338	cytoskeleton organization GO:0007010 (p-value 0.0368)	void
Cerebellum	0.6908	black	301	cellular component organization NA (p-value 0.0152), cellular component organization or biogenesis NA (p-value 0.0264)	void
Cortex	0.8084	brown	862	ensheathment of neurons GO:0007272 (p-value 2.8e-09), axon ensheathment GO:0008366 (p-value 2.8e-09), myelination GO:0042552 (p-value 1.45e-08), neurogenesis GO:0022008 (p-value 3.35e-06), oligodendrocyte differentiation GO:0048709 (p-value 6.76e-06)	Oligodendrocytes in Human brain Module (Geschwind,2010) (p-value 3.216e-141). Oligodendrocytes in Cortex (p-value 3.19e-72). Oligodendrocytes, definite (Cahoy, 2008) (p-value 7.64e-39). Oligodendrocytes from conservative data set (Lein, 2007) (p-value 1.04e-16). Oligodendrocytes-Cahoy (p-value 1.207e-05). Oligodendrocyte-External (p-value 3.665e-46).

Frontal Cortex	0.7939	red	1464	covalent chromatin modification GO:0016569 (p-value 0.000148), organelle organization GO:0006996 (p-value 0.000699), nucleic acid-templated transcription GO:0097659 (p-value 0.00114), nucleobase-containing compound biosynthetic process GO:0034654 (p-value 0.00129), aromatic compound biosynthetic process GO:0019438 (p-value 0.0015)	void
Hippocampus	0.8811	antiquewhite4	339	void	Oligodendrocytes in Human brain Module (Geschwind,2010) (p-value 6.053e-07). Oligodendrocytes in Cortex (p-value 0.008201). Oligodendrocyte-External (p-value 5.488e-10).
Hypothalamus	0.8889	darkred	1218	RNA processing GO:0006396 (p-value 1.25e-10), mRNA processing GO:0006397 (p-value 2.55e-10), mRNA metabolic process GO:0016071 (p-value 7.34e-08), RNA splicing, via transesterification reactions GO:0000375 (p-value 2.69e-07), RNA splicing, via transesterification reactions with bulged adenosine as nucleophile GO:0000377 (p-value 5.95e-07)	void
Nucleus Accumbens	0.8383	sienna3	789	regulation of gene expression GO:0010468 (p-value 9.02e-11), regulation of RNA metabolic process GO:0051252 (p-value 6.55e-10), RNA biosynthetic process GO:0032774 (p-value 7.16e-10), nucleic acid-templated transcription GO:0097659 (p-value 2.34e-09), regulation of nucleobase-containing compound metabolic process GO:0019219 (p-value 2.8e-09)	void
Putamen	0.8211	pink	824	cell cycle process GO:0022402 (p-value 0.00325), organelle fission GO:0048285 (p-value 0.00882), mitotic cell cycle process GO:1903047 (p-value 0.0139), mitotic nuclear division GO:0007067 (p-value 0.0142), nuclear division GO:0000280 (p-value 0.0152)	void
Spinal cord	0.844	plum	556	ensheathment of neurons GO:0007272 (p-value 0.00018), axon ensheathment GO:0008366 (p-value 0.00018), myelination GO:0042552 (p-value 0.00102)	Oligodendrocytes in Human brain Module (Geschwind,2010) (p-value 2.535e-33). Oligodendrocytes in Cortex (p-value 7.784e-13). Oligodendrocytes from conservative data set (Lein, 2007) (p-value 0.01577). Oligodendrocyte-External (p-value 0.001345).
Substantia nigra	0.8884	bisque4	508	carbohydrate phosphorylation GO:0046835 (p-value 0.0145)	void

Supplementary Table 2. Gene co-expression analysis using all 13 human brain region networks sampled by the Genotype Tissue Expression Consortium.
